# Attitudes on voluntary and mandatory vaccination against COVID-19: Evidence from Germany

**DOI:** 10.1371/journal.pone.0248372

**Published:** 2021-05-10

**Authors:** Daniel Graeber, Christoph Schmidt-Petri, Carsten Schröder

**Affiliations:** 1 DIW Berlin / SOEP, Berlin, Germany; 2 Karlsruhe Institute of Technology, Karlsruhe, Germany; 3 Freie Universität Berlin, Berlin, Germany; Middlesex University, UNITED KINGDOM

## Abstract

Several vaccines against COVID-19 have now been developed and are already being rolled out around the world. The decision whether or not to get vaccinated has so far been left to the individual citizens. However, there are good reasons, both in theory as well as in practice, to believe that the willingness to get vaccinated might not be sufficiently high to achieve herd immunity. A policy of mandatory vaccination could ensure high levels of vaccination coverage, but its legitimacy is doubtful. We investigate the willingness to get vaccinated and the reasons for an acceptance (or rejection) of a policy of mandatory vaccination against COVID-19 in June and July 2020 in Germany based on a representative real time survey, a random sub-sample (SOEP-CoV) of the German Socio-Economic Panel (SOEP). Our results show that about 70 percent of adults in Germany would voluntarily get vaccinated against the coronavirus if a vaccine without side effects was available. About half of residents of Germany are in favor, and half against, a policy of mandatory vaccination. The approval rate for mandatory vaccination is significantly higher among those who would get vaccinated voluntarily (around 60 percent) than among those who would not get vaccinated voluntarily (27 percent). The individual willingness to get vaccinated and acceptance of a policy of mandatory vaccination correlates systematically with socio-demographic and psychological characteristics of the respondents. We conclude that as far as people’s declared intentions are concerned, herd immunity could be reached without a policy of mandatory vaccination, but that such a policy might be found acceptable too, were it to become necessary.

## Introduction

Great efforts have been made worldwide to develop a vaccine against COVID-19. When we first drafted this article, in October 2020, 35 different potential vaccines were in clinical trials and 145 were still in the pre-clinical stage. In February 2021, several vaccines have been approved in many countries and are being rolled out, 74 are in clinical trials, and 182 are in the pre-clinical stage [1].

These developments are very encouraging, as a wide availability of vaccines is seen by many as a prerequisite for a return to a “normal” pre-COVID-19 type of social and economic life. With the growing availability of vaccines comes the hope that coercive measures such as restrictions on international trade, contact restrictions, and travel bans, etc., which cause enormous economic and social costs, may soon be removed and will not need to be reimplemented.

Of course, any vaccine is only an effective contribution to a return to normal life if a sufficiently high number of people are actually vaccinated, yielding herd immunity. If so, vaccination secures a public good: protection from COVID-19 for everyone. From a microeconomic perspective, this raises a well-known problem, free-riding: If the vaccination is freely available but not obligatory, then citizens’ individual decisions determine the extent to which this public good is made available. In order to make that decision, they will weigh their own costs and benefits. These costs include the time sacrificed, physical unpleasantness, possible side effects of a vaccination, etc. The benefits to a particular individual are primarily, but not necessarily exclusively, the reduction in risk to that person’s own health or material well-being. From a welfare perspective, if individuals do not take into account the positive externalities on third parties that their own vaccination triggers, there will be an undersupply of the public good. Following [2, 3], individuals’ utility function may also include other-regarding preferences and hence yield a direct benefit from contributions to a public good. In our context, people could therefore benefit from a ‘warm glow of vaccinating’, because by vaccinating themselves they also reduce the risks of others. But even so there is certainly no guarantee that the social optimum will be reached [4] or that a sufficiently high number of people will freely choose to get vaccinated.

It is frequently argued that vaccination should be made mandatory because of the free-rider problem [5]: While vaccinated individuals have incurred private costs in terms of discomfort or money and receive the private benefit of a reduced risk of getting the disease, the major collective benefit, the reduced incidence of disease, is public. If enough other people produce the public benefit, and the circulation of the virus decreases accordingly, an individual might rationally decide to free-ride on others’ decisions. A policy of mandatory vaccination would prevent this.

[6] argue that such a policy would not be necessary: “If vaccinations are perfect, then if one is vaccinated he or she does not care whether others are vaccinated, so there is no longer any public good problem” ([6], p. 70). Hence there would not be a case in favor of mandatory vaccination, as under such a policy, individuals who would have favored not to be vaccinated are made worse off, while those who anyway would get vaccinated are not better off.

However, by definition, ‘perfect’ vaccination means that everyone vaccinated is perfectly immune [6]. In the current situation, it can neither be taken for granted that a perfect vaccination is being or will be provided soon, nor that everyone who wants to also will have the possibility to be vaccinated (both financially and in terms of health). If perfect vaccination is not feasible, however, mandatory vaccination is not dominated by a laissez faire solution [6].

Extensions of this theoretical public good analysis emphasize the relevance of behavioral aspects not typically considered in classical models. The empirical literature also highlights a number of factors that matter for vaccine uptake. For instance [7], show that social norms matter for an individual’s willingness to get a vaccination and that such norms can suppress vaccine uptake even in the presence of frequent disease outbreaks. Further [8], show that the design of public vaccination policies should also take intergroup interactions into account. Other-regarding preferences can explain voluntary vaccination uptake, as argued by [9]. For example [10], show that the presence of individuals who cannot get vaccinated, like babies and the elderly, increases the willingness to get vaccinated. The static model in [6] also does not reflect interactive processes [9, 11]. show that vaccination is the individually best response until a certain vaccination rate is reached in the population and becomes a social dilemma only from this vaccination rate until herd immunity is maximized. Communicating the social benefits of vaccination can have positive effects, particularly when this protects vulnerable groups, but it can also invite free-riding [12]. Those people who cannot get vaccinated themselves for medical reasons are particularly vulnerable: they cannot protect themselves even if they wanted to and, hence, depend on their fellow citizens to protect them by preventing the spread of the virus through their vaccination. Children, too, need to be considered separately. Since they cannot give informed consent to a voluntary vaccination themselves, they might have to be protected from their parents (who might be unwilling to get them vaccinated) in case of particularly serious diseases (see [13, 14]).

There is, in summary, hope that the public goods problem may be overcome, as social and behavioral science offers a wide array of potential policy options to influence people’s perceptions and reactions to the pandemic (for an extensive up-to-date overview, see [15]). It is not clear, however, how the research on well-established vaccines carries over to the current pandemic, and recent developments seem to indicate that the willingness to get vaccinated against the novel coronavirus is currently rather low. We therefore chose to investigate two fundamental questions at the opposite extremes of the spectrum of policy options: would a sufficient number of people voluntarily undergo vaccination to achieve herd immunity? Or would a mandatory vaccination against COVID-19 be acceptable to achieve herd immunity?

A legal duty to be vaccinated against COVID-19 could be an alternative to other coercive measures if one assumes that a high-risk, unregulated, laissez faire approach is not a realistic policy option: it seems irresponsible to lift all restrictions because the virus would soon spread through the entire population. Coercive measures of some kind therefore seem inevitable. Mandatory vaccination could be preferable to other coercive measures, provided the interference with bodily integrity would be considered less socially costly in the long run than the effects of prolonged lockdowns. Emotions run high where vaccination policies are concerned, but because mandatory vaccination might become a realistic scenario, it is worth investigating what the general population thinks about such a policy.

It is important to emphasize that a legal duty to vaccinate against COVID-19 would not imply a legal (or even moral) duty to vaccinate against other diseases. The novel coronavirus is a special case in many respects: In contrast to influenza, for example, the population does not have a background immunity from past infections. In addition, many infected people do not show symptoms (a recent meta-study estimates this to be one in six infected [16]) and, hence, cannot protect others from being infected through voluntary self-quarantining. Thus, people with COVID-19 represent a much higher risk of infection for others than, for example, people who come down with influenza, assuming that these would normally stay at home. Therefore, a vaccination against COVID-19 is much more important from the social perspective than e.g. a vaccination against influenza: not for self-protection, but to protect other people from unintentional infection. Although classic liberal positions (cf. [17]) would reject a paternalist legal obligation to protect oneself through vaccination, they plausibly would favor a policy of mandatory vaccination in the case of COVID-19 to protect others from being harmed. In modern philosophical discussions, even some libertarians are in favor of mandatory vaccination against serious diseases for similar reasons (see [18] and for an overview [19]).

Though there are philosophical reasons supporting a policy of mandatory vaccination, we want to emphasize that we are not advocating it as a concrete policy option for Germany at this moment. Our aim is to understand whether the general public would consider such a policy acceptable, or which sections of the population, and why. To this end, we study the willingness to get vaccinated and the acceptance of a policy of mandatory vaccination against COVID-19 in June and July 2020 in Germany. We use unique real time survey data from a sub-sample (SOEP-CoV) of the German Socio-Economic Panel (SOEP, see [20]). A set of questions about vaccination was part of the later stages of SOEP-CoV, an ongoing research project initiated in April 2020. This so-called ‘vaccination module’ included questions on the willingness to get vaccinated voluntarily and the acceptance of a policy of mandatory vaccination against COVID-19. In addition, individuals could indicate reasons for their preference regarding the second question. Using the rich data of the SOEP, pre-pandemic income, education, household context, personality, political preferences etc., which can be directly linked with SOEP-CoV, we are able to provide a detailed picture on who intends to get vaccinated and who does not.

The most important result of our study is that about 70 percent of adults in Germany would get vaccinated voluntarily against COVID-19 if a vaccine without significant side effects was available. Further, about half of adults in Germany are in favor, and half against, a policy of mandatory vaccination against COVID-19. The approval rate for mandatory vaccination is significantly higher among those who would get vaccinated voluntarily (around 60 percent) than among those who would not get vaccinated voluntarily (27 percent). However, 22 percent of the individuals would disapprove of both a voluntary and a mandatory vaccination and 8 percent can be characterized as ‘passengers’ (they are not willing to get vaccinated but do support a policy of mandatory vaccination, but they might not all be ‘free-riders’ in the standard sense). In this group, surprisingly, 86 percent state that, without a mandatory vaccination, too few individuals would get vaccinated and about 87 percent indicate that most people underestimate how dangerous COVID-19 is. In general, the willingness to get vaccinated is significantly lower for female, younger, and less educated respondents as well as those with lower income. A policy of mandatory vaccination is rejected with higher probability by women and favored by older people and those living in the eastern federal states.

## Data, measures, and methods

### Data: SOEP and SOEP-CoV

The German Socio-economic Panel (SOEP) is among the largest and longest-running representative panel surveys worldwide and is recognized for maintaining the highest standards of data quality and research ethics [20]. In 2020, the survey covers about 30,000 adults in 20,000 households. Since the same individuals and households participate in the study every year, life courses of the respondents can be tracked and intertemporal analyses can be carried out at the individual and at the household level. The data contain information on the respondents’ household situation, education, labor market outcomes, and health, among others (see [20, 21]).

To better understand the effects of the corona pandemic, a special survey called SOEP-CoV was conducted within the framework of the SOEP, which consisted of a random sample of about 6,700 SOEP respondents, (see [21, 22]). SOEP-CoV was surveyed in nine staggered tranches from early April to the end of July 2020 and collected data on the following topics: a) Prevalence, health behavior, and health inequality; b) Labor market and gainful employment; c) Social life, networks, and mobility; d) Mental health and well-being; and e) Social cohesion. Over time, some new question modules were introduced within these five thematic complexes. These included the ‘vaccination module’ (see questionnaires available under www.soep-cov.de/Methodik/).

### Measures: Preferences toward vaccination against COVID-19

The ‘vaccination module’ went into the field with tranches 7 to 9, in June and July 2020, and covered a total of 851 persons aged 19 years and older. At that moment, major research efforts were being undertaken, but it was not clear whether any vaccine would actually be found. The module hence starts with a question on the hypothetical willingness to get vaccinated against COVID-19:

- “Let us assume that a vaccine against the novel coronavirus that is shown to have no significant side effects is found. Would you get vaccinated?”
  The response categories are ’Yes’, ’No’, and ‘no answer’. The module contains a further question about mandatory vaccination with the same response categories:
- “Would you be in favor of a policy of mandatory vaccination against the coronavirus?”
  In addition, the interviewees were asked about their reasons for or against a policy of mandatory vaccination. For this purpose, a filter was used to adapt the arguments according to the respondents’ answers to question (B). The arguments given were as follows:

Argument 1: Others’ willingness to get vaccinated without mandatory vaccination

- Against mandatory vaccination: “Enough people would get vaccinated even without a policy of mandatory vaccination.”
- In favor of mandatory vaccination: “Only with a policy of mandatory vaccination would enough people get vaccinated.”

Argument 2: Misperception of risks

- Against mandatory vaccination: “Most people overestimate the dangerousness of the virus.”
- In favor of mandatory vaccination: “Most people underestimate the dangerousness of the virus.”

Argument 3: Legitimacy of a policy of mandatory vaccinations in general

- Against mandatory vaccination: “A policy of mandatory vaccination is never permissible, even in the case of very dangerous diseases.”
- In favor of mandatory vaccination: “A policy of mandatory vaccinations would make sense also for less dangerous diseases.”

Argument 4: Other reasons (without listing these reasons explicitly)

The first three arguments are of particular relevance for political decision-making. Although there is quite a lot of research on the reasons people have not to get vaccinated themselves, there is much less research on what people think about policies of mandatory vaccinations, and up to present–at least to our knowledge–none on the application to the special case of the novel coronavirus. As the reasons for the individual decision need not carry over to the policy assessment, and given the previously discussed particularities of the coronavirus, we focused on factors that are both of theoretical importance and under discussion in the general public. It would be interesting, for instance, if many people did not have the intention to get vaccinated themselves, yet believed that enough other people would get vaccinated so that mandatory vaccination would not be required. Similarly, it would be surprising if people wanted to get vaccinated yet believed that others overestimated the dangerousness of the virus. Finally, we wanted to see whether people considered mandatory vaccinations potentially legitimate at all.

### Sample selection, weighting, and item non-response

Since SOEP-CoV is a random sample from the SOEP population, the SOEP-CoV data 2020 can be linked with the regular SOEP data of previous years. Thus, attitudes toward vaccination against COVID-19 that were collected during the pandemic can be linked to the characteristics of the respondents before the outbreak of the pandemic (e.g., income or educational level). Since these characteristics were collected before the pandemic, they can be considered unaffected by the pandemic event and, hence, exogenous (S1 File provides definitions of all dependent and independent variables used in the empirical analyses).

The response rate in the vaccination module was high. Altogether, only 4.58 percent of the 851 respondents did not answer the question about voluntary vaccination and 3.41 percent did not answer the question about mandatory vaccination. Of those who supported (objected to) mandatory vaccination, 0.26 (1.82) percent did not provide at least one motive in the follow-up question. Hence, bias from item non-response should be small and we did not correct for it. As the focal variables are coded dichotomously (yes = 1; no = 0), there was no need to remove outliers in them from the database.

To derive population-wide estimates, the SOEP-CoV data is equipped with frequency weights. The weighting of SOEP-CoV follows the standard weighting used in SOEP [23, 24]. Based on the SOEP household weights, weights for all persons in the participating households were generated via a marginal adjustment step and corrected for selection effects. Furthermore, the data were corrected for the fact that some SOEP subsamples were excluded from the SOEP-CoV study from the outset. To address potential selection effects and adjust frequency weights accordingly, we followed the two-step procedure recommended in [25]:

Step 1: Estimation of a logistic regression model where the dependent variable is a dummy variable indicating whether respondents belong to the working sample of tranches 7 to 9 (dummy is equal to one) or not (dummy is zero). All variables included in the following analyses serve as explanatory variables.
Step 2: If at least one analysis variable shows a significant (i.e., *p*-value below 0.05) and at the same time meaningful effect (i.e., coefficient above 0.01) with respect to the assignment to the analysis population, a correction of the SOEP-CoV weights is performed by multiplying the frequency weights by the inverse estimated probability. In other words, multiplying the SOEP-CoV weights belonging to the analysis set by the inverse predicted probability yields the sought adjusted weight that can be used to calculate population statistics. In the present case, an adjustment using the following variables is indicated: Extraversion and whether respondents live in a household in which at least one household member was tested for COVID-19. Overall, selection on observables is very minor. Unless otherwise stated, our results are weighted with the adjusted probability weights.

### Statistical framework

Since the vaccination questions are answered once by each respondent, our empirical strategy is between-person. Uni- and bivariate results for our focal variable, attitudes toward vaccination, are presented as weighted means or percentages. Assessments of differences in attitudes or characteristics between-groups rely on two-tailed t-tests, with statistical significance evaluated at *p*<0.01, *p* <0.05, and *p*<0.10 using the survey weights explained above. Our empirical strategy involves multiple between-group tests. This raises the question of whether a correction is necessary for multiple hypotheses testing. We do not implement such a correction because we seek to compare a certain attitude or characteristic between groups and not to draw, at the end of the test series, a concluding summary of all tests results.

The multivariate analyses to statistically explain vaccination preferences rely on logit regressions. The purpose of the logit regressions is to estimate the probability that a respondent, *i*, with a certain set of characteristics, **X_i_**, will fall into a specific one of the two categories. It can be understood as finding the **β** coefficients that best fit

$$Pr(y_{i}\neq 0|X_{i})=\frac{exp(X_{i}\beta )}{1+exp(X_{i}\beta )},$$ (1)

where *y* is a is a zero-one dummy. We use the logit model to explain attitudes towards both voluntary and mandatory vaccinations. The dummy *y_i_* is set equal to one if a respondent supports voluntary, respectively mandatory, vaccination. Stata fits the logit model using the standard Maximum Likelihood estimator. It considers the binary nature of the outcome variable assuming that a) the logit link function is correct, b) the model is correctly specified, and c) observations are independent. The robust variance estimator which we use below is robust to assumptions a) and b). Since the logit regressions are supposed to describe structures in the data, we do not use sample weights. For all our empirical analyses, we used Stata version 15.

## Results

### Willingness to get vaccinated and attitudes toward a policy of mandatory vaccination

For the questions on voluntary vaccination (A) and mandatory vaccination (B), four groups in the population may be distinguished:

1. Anti-vaccination: interviewees who would not get vaccinated voluntarily against the coronavirus and who also oppose a policy of mandatory vaccination.
2. Anti-duty: interviewees who would get vaccinated voluntarily but oppose a policy of mandatory vaccination.
3. Passengers: interviewees who would not get vaccinated voluntarily but are in favor of mandatory vaccination. We refer to this group as ‘passengers’ because they apparently want to see the public good of herd immunity provided by mandatory vaccination, yet would not voluntarily contribute to this good. Some of these passengers might be free-riders in the standard sense, trying to benefit from the decisions of others while not voluntarily contributing themselves, while others might not be able to get vaccinated for medical reasons. If mandatory vaccination were introduced, the first group, but not the second, would also get vaccinated, of course. Neither group would actually free-ride, but the first might initially have wanted to.
4. Pro-vaccination: interviewees who would get vaccinated voluntarily and are also in favor of mandatory vaccination.

Overall, 70 percent of adults in Germany would voluntarily get vaccinated against the coronavirus, provided a vaccine without significant side effects was available (Table 1: groups 2 and 4). This value corresponds exactly to the results of [26]. From May till September 2020, the COVID-19 snapshot monitoring (COSMO) at the University of Erfurt showed relatively constant values of between 60 and 66 percent; it was only in April that it showed an exceptionally high value of 79 percent, and it has now decreased further (cf. [27], p. 76; an overview of previous studies on the willingness to get vaccinated in Germany is provided in S2 File.). Overall, these studies paint a consistent picture, with a slight decline in the willingness in the second half of 2020.

**Table 1:** Voluntary and mandatory vaccination.

Group	%_*weighted*_	*N_raw_*	Voluntarily?	Mandatory?
**1. Anti-vaccination**	22	173	No	No
**2. Anti-duty**	29	241	Yes	No
**3. Passengers**	8	50	No	Yes
**4. Pro-vaccination**	41	322	Yes	Yes

*Note*. Data from SOEP-CoV. %_*weighted*_ is the weighted share of respondents, *N_raw_* is the unweighted number of observations.

Approximately half of the interviewees are against, and half are in favor of, a policy of mandatory vaccination (against: 51%, groups 1 and 2, in favor: 49%, groups 3 and 4). These values, too, coincide almost exactly with those of the COSMO monitoring since May 2020 (cf. [27], p. 78), which in April showed an approval rate for mandatory vaccination of 73 percent, but later discontinued this question (till July 2020, and it has been decreasing since; see S2 File). The agreement with a policy of mandatory vaccination is clearly higher, namely almost 60 percent (41/(41+29) = 0.59) among those who would get vaccinated voluntarily than with those who would not let themselves be vaccinated voluntarily, i.e. approximately 27 percent (8/(8+22) = 0.27).

### Attitudes toward a policy of mandatory vaccination

The four groups differ noticeably in how they assess the arguments presented to them. This is shown in Table 2, which gives the group-specific approval rate for each argument in combination with the p-values of t-tests in S3.1 Table in S3 File.

**Table 2:** Agreement with specific arguments by group.

Would you get vaccinated voluntarily?	No	Yes
**Arguments against mandatory vaccination**	**1. Anti-vaccination**	**2. Anti-duty**
1. Enough people would get vaccinated even without a policy of mandatory vaccination.	55.6	77.3
2. Most people overestimate the dangerousness of the virus.	53.5	32.1
3. A policy of mandatory vaccination is never permissible, even in the case of very dangerous diseases.	42.7	41.1
For other reasons.	49.5	30.5
**Would you get vaccinated voluntarily?**	**No**	**Yes**
**Arguments in favor of mandatory vaccination**	**3. Passengers**	**4. Pro-vaccination**
1. Only with a policy of mandatory vaccination would enough people get vaccinated.	86.1	92.3
2. Most people underestimate the dangerousness of the virus.	86.8	81.8
3. A policy of mandatory vaccinations would make sense also for less dangerous diseases.	64.7	73.1
For other reasons.	17.8	20.5

*Note*. Data from SOEP-CoV. All numbers in percent and weighted.

#### Argument 1

The groups differ markedly in how likely they think it is that others will get vaccinated. Among the two groups that are against a policy of mandatory vaccination, 56 percent of the ‘anti-vaccination’ group (who would not get vaccinated voluntarily) think that their fellow citizens would get vaccinated sufficiently frequently such that mandatory vaccinations would not be necessary. Almost 80 percent of the ‘anti-duty’ group (the members of which would get vaccinated voluntarily) think the same. Among the two groups that are in favor of mandatory vaccination, 85 percent of the ‘passengers’ (who would not voluntarily get vaccinated) think that the others would not voluntarily get vaccinated either, as do slightly more than 90 percent of the ‘pro-vaccination’ group (who would also get vaccinated voluntarily).

#### Argument 2

These results run in parallel with the assessment of the dangerousness of the virus. Even though the analysis is not causal, we can see that about 50 percent of the ‘anti-vaccination’ group and 30 percent of the ‘anti-duty’ group think that most people overestimate the dangerousness of SARS-CoV-2. Exactly the opposite, that most people underestimate the dangerousness, is believed by nearly 90 percent of the ‘passenger’ group and by slightly more than 80 percent of the ‘pro-vaccination’ group. Summarizing the numbers differently, one could say that groups 2 and 4, who would voluntarily get vaccinated, probably have similar opinions about whether their fellow citizens correctly assess the danger posed by the virus. About 80 percent of the members of the ‘pro-vaccination’ group think that most people underestimate the danger. Of the members of the ‘anti-duty’ group, we only know with certainty that 30 percent of them believe that most people overestimate the danger–we do not know, however, how the remaining 70 percent are divided between ’underestimate’ and ’correctly estimate’. The difference between the corresponding values for groups 1 and 3 is significantly higher.

Looking at arguments 1 and 2, we may conclude that there is a high level of disagreement among the population about the dangerousness of the virus. This disagreement probably explains why people have such different attitudes toward getting vaccinated and toward the necessity (or not) of a policy of mandatory vaccination.

The position of the group of the ‘passengers’ is hard to understand. On the one hand, they favor a policy of mandatory vaccination, presumably because, as they do believe, the dangerousness of the virus is often underestimated. On the other hand, they probably assume that they themselves do not underestimate that dangerousness, but nevertheless would not get vaccinated voluntarily. One reason for this could be their medical condition: they might be willing but unable to get vaccinated for medical reasons. If so, they would not be trying to free-ride. It is unclear, however, how much weight the appeal to such a hypothetical medical contraindication should have, given that at the time of the interviews, no vaccine was even available. Some, but not all, of the ‘passengers’ are probably free-riders in the standard sense.

#### Argument 3

Approximately 40 percent of both the ‘anti-vaccination’ group and the ‘anti-duty’ group agree with the statement that a mandatory vaccination is never permissible, not even with very dangerous diseases. Since these two groups reject mandatory vaccination against COVID-19, this means that for the remaining 60 percent of the group, mandatory vaccination may well be permissible–but apparently only for diseases that they would have to consider as even more dangerous than COVID-19. Conversely, well over 60 percent of the ‘passenger’ group and just over 70 percent of the ‘pro-vaccination’ group agree with the statement that a policy of mandatory vaccination would also make sense for less dangerous diseases. In combination with the results for argument 2, these two groups could therefore believe that their fellow citizens also underestimate the danger of such other diseases. It is interesting to note that, overall, people in Germany estimate the probability that the novel coronavirus will cause a life-threatening disease within the next twelve months to be high (cf. [20]). This probability is around 25 percent across our four groups. In group 1 it is 20 percent, in group 2 around 27 percent, in group 3 it is 30 percent and in group 4 it is 25 percent (see Table 3).

**Table 3:** Characteristics by group.

Groups:		1	2	3	4
Characteristics	(all groups)	Anti-vacc.	Anti-duty	Passen-ger	Pro-vacc.
Percentage female (%)	43.95	58.2	37.49	65.88	36.35
Age	52.77	47.58	49.39	55.59	57.43
Tertiary education (2017/18, %)	27.66	11.65	36.46	11.64	33.7
Net household income pm, 1k Euro (2018/19)	3.04	2.84	3.34	2.29	3.09
Share with Children below age 17 (2019, %)	24.82	26.94	22.65	21.69	25.79
Share living in East Germany (%)	18.93	16.53	13.32	19.68	24.04
Extraversion (2019)	0.02	0.27	-0.18	0.02	0.01
Conscientiousness (2019)	-0.05	-0.1	-0.1	0.13	-0.01
Openness to experience (2019)	-0.02	-0.33	0.15	0.07	0.01
Neuroticism (2019)	-0.07	0.08	0.04	-0.22	-0.2
Agreeableness (2019)	-0.01	-0.2	-0.03	0.36	0.05
Willingness to take risks (2019)	0.07	0.11	0.09	-0.28	0.11
Health: Self-Assessment	0.01	-0.16	0.02	0.37	0.01
Number of risky diseases (2019)	0.93	0.59	0.82	1.5	1.08
Test for COVID-19 in household (%)	7.29	7.66	5.27	15.01	6.97
Positive test for COVID-19 in household (%)	0.31	1.39	0	0	0
Prob. of life-threatening disease (in %)	24.51	19.93	26.58	30.08	24.51
Political preference	-0.12	-0.33	-0.01	-0.02	-0.11

*Note*. Data from SOEP and SOEP-CoV. All numbers in percent and weighted. In column “Characteristics” indicate data surveyed in years different from year 2020. The Big Five, risk taking, self-assessed health, and political orientation are measured in standard deviations. For political preferences, higher values are associated with a left political orientation. S1 File provides definitions of all the variables and details on the construction of the Big-5.

**Other reasons** (which were not further broken down in the questionnaire for capacity reasons) are important primarily among those respondents who would not themselves get vaccinated and also oppose mandatory vaccination. Although questions (A) and (B) explicitly assume that a vaccine would not have any significant side effects, this could be due to a deeper skepticism about vaccination, which we hope to be able to explore in future research (on ’vaccine denialism’ see [28]).

### Characteristics of the ‘anti-vaccination’, ‘anti-duty’, ‘passenger’, and ‘pro-vaccination’ groups

#### Description of the individual characteristics of the groups

We would like to know in more detail who is in favor of a policy of mandatory vaccination against COVID-19 and who is opposing it, as well as what the socio-economic characteristics of those who would get vaccinated and of those who would not are. Table 3 shows how the four groups defined above differ across various socio-demographic characteristics (measured before the pandemic), personality (measured before the pandemic), health (before and during the pandemic), and political orientation (measured before the pandemic). Statistical t-tests for the significance of differences in characteristics between groups are shown in S3.2 Table in S3 File assuming equal variances across groups. S3.3 Table in S3 File provides supporting evidence: tests for equality of variance across groups provides support for this assumption in about 90% of the cases, and as S3.4 Table in S3 File. shows, relaxing the equality of variances assumption does not change our conclusions.

*Socio-demographic characteristics*. Almost 60 percent of the ‘anti-vaccination’ group are female, they are on average 48 years old, 12 percent of them have a university degree and their monthly net household income in 2019 averaged just under EUR 2,800. Around 27 percent have children under 16 and around 17 percent live in the eastern German states. ‘Passengers’ do not differ in their characteristics statistically significantly from this group. The members of the ‘anti-duty’ group, by contrast, are much more likely to be male and more often have a university degree. In comparison to the ‘anti-vaccination’ group, the members of the ‘pro-vaccination’ group are also more often male and older, and are also more likely to have a university degree. In particular, older interviewees are more likely to be in groups that favor mandatory vaccination and persons with a university education in groups comprising those who would get vaccinated voluntarily.

*Personality traits*. SOEP collects the personality traits of the respondents using a battery of questions that measure the five dimensions of the so-called ’Big Five’ [29]. The Big Five are the five most important groups of character traits in personality research: ’openness’, ’conscientiousness’, ’extraversion (sociability)’, ’tolerance’, and ’neuroticism’. Furthermore, risk attitude is surveyed. We see that members of the ‘anti-vaccination’ group tend to be more sociable but less open than the other groups. Their willingness to take risks is similar to that of the members of the ‘anti-duty’ and of the ‘pro-vaccination’ groups, but is significantly higher than that of ‘passengers’. Members of the ‘anti-duty’ group are particularly unsociable compared to the other groups, but open to new experiences. The ‘passengers’ are, like the members of the ‘pro-vaccination’ group, less neurotic. They are particularly tolerable and the least willing to take risks of the four groups.

*Health*. As far as the health of those surveyed is concerned, statistically significant differences are only evident in the number of illnesses: Members of the ‘anti-vaccination’ group have significantly fewer risk diseases than ‘passengers’ and members of the ‘pro-vaccination’ group. ‘Anti-duty’ members, on the other hand, have significantly fewer diseases than the ‘passengers’. Thus, overall, it may be said that those who refuse a policy of mandatory vaccination have fewer risk diseases at the time of the survey. There are no differences between the groups in terms of whether a member of the respondent’s household has already undergone a test for an infection with corona. It should be noted, however, that the number of cases of those tested for an infection is comparatively small.

*Political orientation*. As far as the political orientation of the respondents is concerned, no systematic significant differences between the four groups are identified. Only the members of the ‘anti-vaccination’ group seem to be positioned somewhat more to the right in the party spectrum than the members of the ‘anti-duty’ group.

#### Multivariate description of the characteristics of the four groups

The differences and similarities with regard to group composition described above always refer to a single characteristic, i.e. they are univariate. Additionally, the relationships between individual characteristics of the respondents–after taking other characteristics into account–and their attitude toward mandatory or voluntary vaccination are explained below using a multivariate model (logistic estimation; see Eq (1)). The dependent variable is either an indicator that describes the respondent’s own willingness to get vaccinated voluntarily (value 1 = yes; 0 = no; Table 4) or an indicator (value 1 = yes; 0 = no) that describes whether the respondents favors a policy of mandatory vaccination (Table 5). As our interest is the explanation of data structures, we do not use survey weights in the multivariate analysis.

**Table 4:** Average marginal effects of individual characteristics on willingness to get vaccinated (N = 678, Pseudo R2 of underlying Logit estimation: 0.103).

Explanatory variable	Effect	S.E.	LB 95% CI	UB 95% CI	z-statistic	p-value
Female	-0.100	0.035	-0.170	-0.031	-2.848	0.004
Age	0.004	0.001	0.001	0.006	2.738	0.006
Tertiary education	0.131	0.036	0.061	0.201	3.647	0.000
Net monthly income per household, 1k EUR	0.025	0.013	0.001	0.050	2.007	0.045
Children younger than 17	-0.004	0.040	-0.082	0.073	-0.104	0.917
Eastern federal states	0.002	0.040	-0.076	0.079	0.043	0.966
Extraversion	-0.010	0.017	-0.044	0.023	-0.608	0.543
Conscientiousness	-0.019	0.018	-0.055	0.016	-1.079	0.281
Openness to experience	0.030	0.018	-0.006	0.066	1.647	0.100
Neuroticism	-0.024	0.019	-0.062	0.013	-1.285	0.199
Agreeableness	-0.021	0.017	-0.055	0.013	-1.202	0.229
Willingness to take risks	-0.025	0.018	-0.061	0.011	-1.373	0.170
Health: Self-assessment	-0.006	0.018	-0.041	0.030	-0.315	0.753
Number of risk diseases	0.017	0.019	-0.020	0.055	0.903	0.366
Test for COVID-19 in household	-0.048	0.051	-0.148	0.052	-0.948	0.343
Positive test for COVID-19 in household	-0.319	0.304	-0.915	0.277	-1.050	0.294
Prob. of life-threatening disease (in %)	0.003	0.001	0.001	0.004	3.252	0.001
Political preferences	0.012	0.016	-0.020	0.044	0.745	0.456

*Note*. Data from SOEP and SOEP-CoV. All numbers unweighted. Column “Explanatory variable” indicates data surveyed in years different from year 2020. S.E. denotes standard error. LB denotes lower and UB upper bound of the confidence band (CI). S1.1 Table in the S1 File provides definitions of all the variables. Marginal effects. The Big Five, risk taking, self-assessed health and political orientation are measured in standard deviations. For political preferences, higher values are associated with a left political orientation. S1 File provides definitions of all the variables and details on the construction of the Big-5.

**Table 5:** Average marginal effects of individual characteristics on attitudes toward mandatory vaccinations (N = 682, Pseudo R2 of underlying Logit estimation: 0.079).

Explanatory variable	Effect	S.E.	LB 95% CI	UB 95% CI	z-stat.	p-value
Female	-0.094	0.039	-0.172	-0.017	-2.395	0.017
Age	0.006	0.001	0.003	0.009	3.934	0.000
Tertiary education	-0.049	0.042	-0.132	0.033	-1.165	0.244
Net monthly income per household, 1k EUR	0.004	0.011	-0.017	0.025	0.380	0.704
Children younger than 17	0.038	0.046	-0.053	0.128	0.810	0.418
Eastern federal states	0.144	0.046	0.054	0.234	3.146	0.002
Extraversion	0.001	0.019	-0.037	0.038	0.035	0.972
Conscientiousness	0.014	0.020	-0.025	0.053	0.705	0.481
Openness to experience	-0.001	0.019	-0.039	0.036	-0.073	0.942
Neuroticism	-0.044	0.020	-0.084	-0.005	-2.206	0.027
Agreeableness	-0.002	0.019	-0.040	0.035	-0.110	0.913
Willingness to take risks	0.010	0.020	-0.029	0.049	0.498	0.619
Health: Self-assessment	-0.002	0.021	-0.043	0.038	-0.115	0.908
Number of risk diseases	0.022	0.020	-0.018	0.062	1.068	0.285
Test for COVID-19 in household	0.007	0.058	-0.106	0.121	0.126	0.900
Positive test for COVID-19 in household	n.a.	n.a.	n.a.	n.a.	n.a.	n.a.
Prob. of life-threatening disease (in %)	0.003	0.001	0.001	0.005	3.176	0.001
Political preferences	0.002	0.018	-0.034	0.038	0.120	0.905

*Note*. Data from SOEP and SOEP-CoV. All numbers unweighted. Column “Explanatory variable” indicates data surveyed in years different from year 2020. S.E. denotes standard error. LB denotes lower and UB upper bound of the confidence band (CI). “Positive tests for COVID-19 in household” does not exhibit variation when including the set of other controls. S1.1 Table in the S1 File provides definitions of all the variables. Marginal effects. The Big Five, risk taking, self-assessed health and political orientation are measured in standard deviations. For political preferences, higher values are associated with a left political orientation. S1 File provides definitions of all the variables and details on the construction of the Big-5.

With regard to the willingness to voluntarily get vaccinated (Table 4), some significant differences in socio-demographic characteristics are observed. If all other characteristics are kept constant, the willingness to vaccinate is about 10 percentage points lower in women than in men. It is positively associated with age (0.4 percentage points per year of life), education (13 percentage points if respondents have a university degree compared to the other education categories), and household income (2.5 percentage points per 1,000 euros). The personality traits of the Big Five do not correlate with the respondents’ willingness to vaccinate; only openness is slightly positively associated with the willingness to vaccinate. In the health block, there is also only one significant variable that correlates with the willingness to get vaccinated: The higher the respondents estimate the probability that the virus could trigger a life-threatening disease in them, the more willing they are to be vaccinated.

A policy of mandatory vaccination (Table 5) is also rejected with higher probability by women, but favored by older people and those living in the eastern federal states, *ceteris paribus*. Approval is negatively associated with neuroticism, i.e. emotional instability, and positively associated with the subjective probability of contracting life-threatening COVID-19.

The tables presenting the logit estimations include an initial model diagnostic: the Pseudo-*R*^2^. In S4 File, we present two additional model diagnostics: First, the linktest for both logit models does not find any evidence for model misspecifications. Second, a receiver operator characteristic (ROC) analysis provides evidence that the predictive power of our two models is acceptable. In addition, to assess multicollinearity, we have computed variance inflation factors (VIF) in S5 File. As a rule of thumb, a variable whose VIF values exceeds 10 may merit further investigation. In both regressions, the VIF of none of the explanatory variable exceeds 7.7 and the average VIF over all variables is below 2.1. It should also be noted that the two separate logit models do not model correlation and heteroscedasticity between the two outcomes (vaccinate voluntarily or obligatorily). Hence, in S6 File, as a robustness check, we have estimated a multivariate probit model using Stata’s mvprobit command that relies on simulated maximum likelihood [30]. S6.1-S6.6 Tables in S6 File compare the coefficients of the multivariate probit with the two separate models. Overall, there are some changes in the magnitude of the coefficients, but no changes in the signs of the regression coefficients or significance levels.

It is possible that respondents who did not give an answer about their vaccination preferences–for example, because they are still undecided–would decide to vaccinate or support mandatory vaccination after an adequate vaccination campaign. In a robustness check, we followed this argument by assigning respondents who refused to answer the question about voluntary or mandatory vaccination to the ‘yes’ category and repeated the logit estimation. This does not change our results (see S7.1 and S7.2 Tables in S7 File, S8 File provides our Stata code, which prepares the data and conducts all the statistical analyses, as well as the outputs of the multivariate estimations).

Finally, Table 6 provides a statistical comparison of the marginal effects from the model on willingness to get vaccinated (Table 4) and attitudes toward mandatory vaccinations (Table 5). We find no significant differences in marginal effects between the two models except for two variables: tertiary education and eastern federal states. The marginal effect for tertiary education is significantly larger for the willingness to get vaccinated model while the opposite is true for eastern federal states.

**Table 6:** Comparing average marginal effects across models (N = 680).

Explanatory variable	Effect voluntary	Effect mandatory	Chi2(1)	p-value
Female	-0.100	-0.094	0.013	0.909
Age	0.004	0.006	1.343	0.247
Tertiary education	0.131	-0.049	10.588	0.001
Net monthly income per household, 1k EUR	0.025	0.004	1.634	0.201
Children younger than 17	-0.004	0.038	0.468	0.494
Eastern federal states	0.002	0.144	5.538	0.019
Extraversion	-0.010	0.001	0.189	0.664
Conscientiousness	-0.019	0.014	1.559	0.212
Openness to experience	0.030	-0.001	1.408	0.235
Neuroticism	-0.024	-0.044	0.528	0.468
Agreeableness	-0.021	-0.002	0.531	0.466
Willingness to take risks	-0.025	0.010	1.671	0.196
Health: Self-assessment	-0.006	-0.002	0.014	0.904
Number of risk diseases	0.017	0.022	0.028	0.867
Test for COVID-19 in household	-0.048	0.007	0.519	0.471
Prob. of life-threatening disease (in %)	0.003	0.003	0.010	0.919
Political preferences	0.012	0.002	0.161	0.688

*Note*. Data from SOEP and SOEP-CoV. All numbers unweighted. The first two columns display average marginal effects of the characteristics on the willingness to get vaccinated and attitudes towards mandatory vaccination. The third column displays the Chi-squared statistic of a comparison of the two estimates and the fourth column displays the associated p-value. The S1 File provides definitions of all the variables and details on the construction of the Big-5. The Big Five, risk taking, self-assessed health and political orientation are measured in standard deviations. For political preferences, higher values are associated with a left political orientation.

## Discussion

Politicians must make decisions which are based on incomplete information yet have far-reaching consequences for public health, personal freedom, and economic prosperity. It seems that many citizens are prepared to behave responsibly in the sense that they are prepared to endure a ‘little sting’ for the good of all: a vast majority of the German population (70 percent) state that they would get vaccinated as soon as a vaccine against COVID-19 was available. This means that under favorable conditions, a legal duty to get vaccinated to achieve herd immunity might not be necessary. It should, however, be noted that the question was asked in a stylized context: Potential side effects or ineffectiveness of the then hypothetical vaccine were assumed away. Though there is no reason to believe the vaccines currently being administered are more problematic in this respect than any other vaccines in use, neither can strictly be guaranteed in reality. In addition, the time required for a vaccination, the process of the vaccination itself (i.e. the injection), bureaucratic administration (e.g. making an appointment with the family doctor) or any necessary co-payment should *de facto* reduce the willingness to get vaccinated. Furthermore, at the time of writing, not only is it still unclear how quickly a vaccine can even be produced in the quantity required and administered to enough people, it is also unclear how long its effect will last. It is not even clear what percentage of the population would have to be vaccinated to achieve herd immunity, as this also depends on individual behavior and legal (or ethical) norms which are likely to continue to change (e.g. an explicit or implicit obligation to wear a mask of a specific variety in public transport or a testing obligation for people returning from trips abroad) [31, 32]. Hence, a sufficiently high willingness to get vaccinated in the ‘best case’ scenario investigated here is an idealization and in any case only one relevant factor among many.

We observed there to be gender differences in the willingness to get vaccinated: women are less willing to get vaccinated, and also less willing to support a policy of mandatory vaccination. This is surprising, given that men are generally less likely to engage in preventive behavior [33] and women have been shown to be more willing to engage in preventive behavior in the pandemic, for instance by wearing face masks when recommended [34], and they also seem to be more compliant with other measures in general [35]. However, men are also more severely affected by the coronavirus [36] and women generally more skeptical about vaccinations, especially against COVID-19 [37]. We don’t know whether our interviewees frame their decision to get vaccinated or not as a situation of a social dilemma, but if so, previous results on gender differences in cooperation suggest men and women might have to be addressed differently to influence their decisions [38, 39]. We also observed that income and education increase the willingness to get vaccinated voluntarily. It has also been shown recently that the willingness to pay for a vaccine against Covid-19 is positively impacted by, among other variables, income [40].

A mandatory vaccination would almost certainly achieve herd immunity against COVID-19, since all those for whom there is no medical contraindication would also get vaccinated. About half of the respondents approve and disapprove, respectively, of such a mandatory vaccination policy. In this context, the strong disagreement among the participants of the study regarding the dangerousness of the virus is particularly striking. Many of those who reject a policy of mandatory vaccination assume the dangerousness of the virus is being overestimated by others, while those who approve of a policy of mandatory vaccination seem to believe the exact opposite. This is highly problematic: at most one of the two groups can be right. Plausibly, the interviewees themselves differ in how dangerous they think the virus is. This yields a concrete and important policy recommendation (see also [40, 41]): we need more reliable data on the dangerousness of SARS-CoV-2 and to communicate this data more clearly to the general public. Though the ‘knowledge-deficit’ explanation of low vaccine uptake might not work for well-established vaccines [42], we have found evidence that this might be different for COVID-19.

We are not recommending a policy of mandatory vaccination in this paper, but merely investigating the attitudes of people towards it. A policy of mandatory vaccination would be an extreme solution to solve the potential problem of low vaccine uptake, and a lot may be said in favor of less extreme policies (as outlined in [15], for instance). Vaccination could also be made mandatory only for certain groups of people (e.g. nurses, physicians, physiotherapists, people working in confined spaces, people travelling on public transport etc.), or only after time has conclusively shown that not enough people actually get vaccinated. It might also turn out that people are unwilling to take the second dose of a two-dose vaccine, or not accept refresher doses, which would further complicate the situation and might require subtle intertemporal strategy choice. Before making any vaccination mandatory, people could also be paid or incentivized in other ways to accept it [43]. If, as we hope, people take the external effects of their action into account, and a sufficiently high number of people get vaccinated as a result, mandatory vaccination won’t be necessary.

## Conclusion

This article investigates the willingness to get vaccinated and the acceptance of a policy of mandatory vaccination against COVID-19 in June and July 2020 in Germany. Our first main result is that a large majority of about 70 percent of adults in Germany would voluntarily get vaccinated against the novel coronavirus if a vaccine without side effects was available. Our second result is that about half of this population is in favor of, and half against, a policy of mandatory vaccination. Our third main result is that the individual willingness to get vaccinated and acceptance of a policy of mandatory vaccination correlates systematically with several socio-demographics (gender, age, education, income) but, overall, not with psychological characteristics of the respondents.

When interpreting the results from our survey, it should be noted that preferences were elicited in an ideal-typical situation: a vaccine which is effective and free of side effects is immediately available for the entire population at zero cost. Future research will have to show how actual vaccination behavior differs in real-life situations that deviate from this ideal-typical situation.

## Supporting information

S1 File Variable definitions. (DOCX)

S2 File Comparison with further studies in Germany. (DOCX)

S3 File Complementary estimation results. (DOCX)

S4 File Additional diagnostics for the logit models. (DOCX)

S5 File Multicollinearity across explanatory variables. (DOCX)

S6 File Functional form assumptions and simultaneity. (DOCX)

S7 File Imputation. (DOCX)

S8 File Stata code. (DOCX)
