# Supplementary material for: Attitudes on voluntary and mandatory vaccination against COVID-19: Evidence from Germany
Source: PLoS One. 2021 May 10;16(5):e0248372. doi: 10.1371/journal.pone.0248372 (PMC8109805; doi:10.1371/journal.pone.0248372)
Supplement: S1 File — (DOCX) [file pone.0248372.s001.docx]

# S1 File: Variable Definitions

**S1.1 Table:** Variable definitions

| Variable | Description | Year |
| --- | --- | --- |
| Female | Indicator variable:  1: female respondents  0: else | 2019 or 2018 |
| Age | Age in years | 2018 |
| Tertiary education | Indicator variable:  1: respondents with tertiary education (categories 8 and 9 in CASMIN-Scale)  0: else | 2018 or 2017 |
| Monthly household net income (prices 2015) | Self-reported value from year 2019 if available; else from 2018 or generated household income | 2019 or 2018 |
| Children younger than 17 | Indicator variable:  1: at least one child below age 17 living in household  0: else | 2019 |
| Eastern Federal States | Indicator variable:  1: living in Eastern Federal States  0: else | 2020 |
| Extraversion | First factor of principle component analysis of BIG-5 item battery | 2019 |
| Conscientiousness | Second factor of principle component analysis of BIG-5 item battery | 2019 |
| Openness to experience | Third factor of principle component analysis of BIG-5 item battery | 2019 |
| Neuroticism | Fourth factor of principle component analysis of BIG-5 item battery | 2019 |
| Agreeableness | Fifth factor of principle component analysis of BIG-5 item battery | 2019 |
| Willingness to take risk | Risk tolerance, measured on 11-point Likert scale: 0 "unwilling to take risk"; 10 "very willing to take risk" | 2019 |
| Health: Self-assessment | Self-assessed health status, measured on 5-point Likert scale: 1 "very good"; 5: "bad" | 2019 or 2018 |
| Number of risky diseases | Number of medical diseases associated with complicated COVID-19 infection: Diabetes, asthma, heart disease, stroke, high blood pressure, dementia, joint diseases (such as rheumatism) and obesity | 2019 or 2018 |

| Test for COVID-19 in household | Indicator variable:  1: at least one household member was tested  0: else | 2020 |
| --- | --- | --- |
| Positive test for COVID-19 in household | Indicator variable:  1: at least one household member was positively tested  0: else | 2020 |
| Prob. of life-threatening disease | Self-assessed probability that COVID-19 causes a life-threatening disease | 2020 |
| Political preference | Political preference:  -1: respondent selected values 6 to 10 on a scale from 0 "very left wing" to 10 "very right wing"  0: respondent selected value 5 on 0 to 10 scale or did not answer  1: respondent selected values 0 to 4 on 0 to 10 scale | 2019 |

**Construction of Big 5 personality traits**

In SOEP, each dimension of the Big Five is surveyed using three to four items. Items with negative polarity are recoded so that they have a positive polarity. This means that higher levels of items are associated with a higher level of the underlying personality trait. A subsequent principal component analysis of this recoded item battery with subsequent Varimax rotation confirms that the 16 items actually charge on five different factors. The item battery implemented in SOEP is as follows, with items with negative polarity marked with "-":

Openness

1. is original, brings in new ideas

2. appreciates artistic, aesthetic experiences

3. has a vivid imagination

4. is inquisitive

Scale: 1 (Not at all / not applicable at all) to 7 (fully applies)

Conscientiousness

1. works thoroughly

2. is rather lazy (-)

3. performs tasks effectively and efficiently

Scale: 1 (does not apply at all) to 7 (fully applies)

Extraversion

1. is communicative, talkative

2. can come out of himself, is sociable

3. is reserved (-)

Scale: 1 (does not apply at all) to 7 (fully applies)

Agreeableness

1. is sometimes a little rough with others (-)

2. can forgive

3. is considerate and friendly with others

Scale: 1 (does not apply at all) to 7 (fully applies)

Neuroticism

1. is often worried

2. becomes slightly nervous

3. is relaxed, can handle stress well (-)

Scale: 1 (does not apply at all) to 7 (fully applies)
