# Supplementary material for: Attitudes on voluntary and mandatory vaccination against COVID-19: Evidence from Germany
Source: PLoS One. 2021 May 10;16(5):e0248372. doi: 10.1371/journal.pone.0248372 (PMC8109805; doi:10.1371/journal.pone.0248372)
Supplement: S2 File — (DOCX) [file pone.0248372.s002.docx]

# S2 File: Comparison with Further Studies in Germany

In the main body of the paper, SOEP-CoV is used to compute two key figures:

1. the share of adult persons in Germany that are willing to get vaccinated against the novel coronavirus; and
2. the share of adult persons in Germany that are in favor of a policy of mandatory vaccination against the novel coronavirus.

First, we compare our estimate of the population share that is willing to get a vaccination with estimates from other studies in Germany. S2.1 Table provides basic information about these studies. Apart from the first wave of YouGov for the German news agency ‘Deutsche Presse Agentur’ (dpa) (N=2,056), all surveys had sample sizes of around 1,000. Most studies had one or two waves. An exception is COSMO with 21 waves being conducted between April and December 2020.

All the comparisons have to be done with caution for two reasons. First, the respective questions are formulated differently across the surveys. In particular, while most surveys ask about the willingness to get vaccinated without making any assumptions about the vaccine, two surveys explicitly assumed positive attributes (e.g. no side effects), which is likely to lead to higher acceptance rates. Second, response categories differ. Possible options include dichotomous (Yes/No) or more granular categories.

**S2.1 Table:** List of surveys studying the willingness to get vaccinated against the novel coronavirus in Germany in 2020

| **Source** | **Wave** | **Start** | **End** | **Sample size** | **Assumptions about vaccine** | **Share of agreement (%)** |
| --- | --- | --- | --- | --- | --- | --- |
| ARD-DeutschlandTREND | 1 | Aug 2 | Aug 4 | 1011 | - | 74 |
| ARD-DeutschlandTREND | 2 | Nov 9 | Nov 10 | 1004 | - | 71 |
| ZDF-Politbarometer | 1 | Nov 24 | Nov 26 | 1330 | - | 51 |
| KANTAR | 1 | May 28 | June 1 | 1000 | - | 67 |
| KANTAR | 2 | Nov 10 | Nov 14 | 1000 | - | 67 |
| HCHE COVID-19 study | 1 | Apr 2 | Apr 15 | ca. 1000 | - | 70 |
| HCHE COVID-19 study | 2 | June 9 | June 22 | ca. 1000 | - | 61 |
| HCHE COVID-19 study | 3 | Sept 19 | Nov 5 | ca. 1000 | - | 57 |
| HCHE COVID-19 study | 4 | Nov 5 | Nov 16 | ca. 1000 | - | 57 |
| University of Heidelberg | 1 | June 30 | July 7 | 1351 | - | 54.7 |
| University of Heidelberg | 2 | Nov 30 | Dec 11 | 1099 | - | 46 |
| YouGov for dpa | 1 | May 26 | May 28 | 2056 | - | 49 |
| YouGov for dpa | 2 | Dec 22 | Dec 23 | - | - | 65 |
| SOEP-CoV | 1 | June 8 | July 4 | 850 | No significant side effects | 70 |
| COSMO | 1-21 | Apr 14 to Dec 15  (1 day each) | | ca. 1000 each | Effective, secure and recommended | 79 to 49 |

*Note*: Dashes indicate that no information was available on the given item. For wave 2 of YouGov, the exact survey period is not known, we assume it was two days before publication of the results in the press. Note also that the percentage figure for COSMO gives the share of persons answering, on a scale from 1 to 7, a number between 5 and 7. It is the only study considered using a numerical scale for the answers.

S2.1 Fig summarizes the population share that would voluntarily get a vaccine. It conveys two messages. First, between April and December 2020, the willingness to get vaccinated declines. Only one study (YouGov) indicates the opposite. Second, the population share from SOEP-CoV, collected between June and July, is similar to other surveys for that time.

**S2.1 Fig:** Results from surveys studying the willingness to get vaccinated against the novel coronavirus in Germany


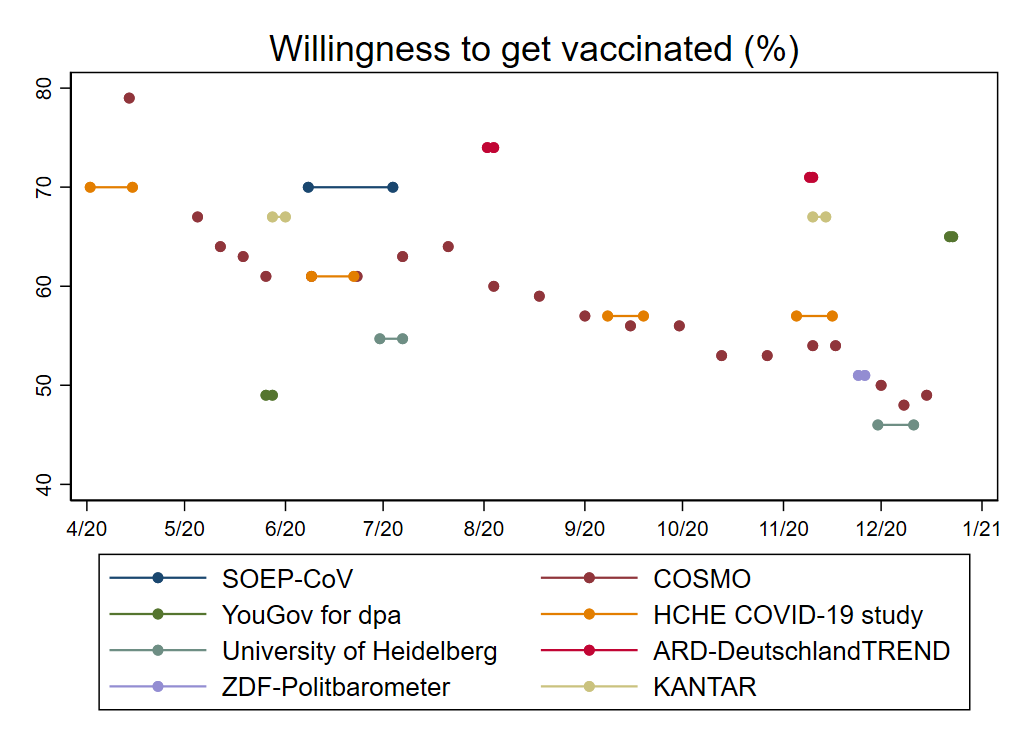


*Note*: Lines indicate the survey periods. If there are no lines, surveys were performed on a single day.

Regarding mandatory vaccination, S2.2 Table shows that we have fewer surveys to compare than in the case of the willingness to get vaccinated. While YouGov and SOEP-CoV posed questions and answer options almost identically (yes/uncertain/no), COSMO gives the share of respondents giving an answer with positive tendency (between 5 and 7 on a scale from 1 to 7). S2.2 Fig shows that the studies show similar levels of acceptance of a policy of mandatory vaccination. SOEP-CoV is almost exactly in line with the finding from COSMO in June/July 2020.

**S2.2 Table:** List of surveys studying the acceptance of a policy of mandatory vaccination against the novel coronavirus in Germany (in 2020 unless stated otherwise)

| **Source** | **Wave** | **Start** | **End** | **Sample size** | **Share of agreement (%)** |
| --- | --- | --- | --- | --- | --- |
| YouGov for dpa | 1 | May 26 | May 28 | 2056 | 44 |
| YouGov for dpa | 2 | Jan 4, 2021 | Jan 5, 2021 | - | 33 |
| SOEP-CoV | 1 | June 8 | July 4 | 850 | 50 |
| COSMO | 1 - 3, 7 - 21 | April 14 to Dec 15  (one day each) | | ca. 1000 each | 55 to 36 |

*Note*: Dashes indicate that no information was available on the given item. For wave 2 of YouGov, the exact survey period is not known, we assume it was two days before publication of the results in the press.. Note also that the percentage figure for COSMO gives the share of persons answering, on a scale from 1 to 7, a number between 5 and 7. It is the only study considered using a numerical scale for the answers.

**S2.2 Fig:** Results from surveys studying the acceptance of a policy of mandatory vaccination against the novel coronavirus in Germany


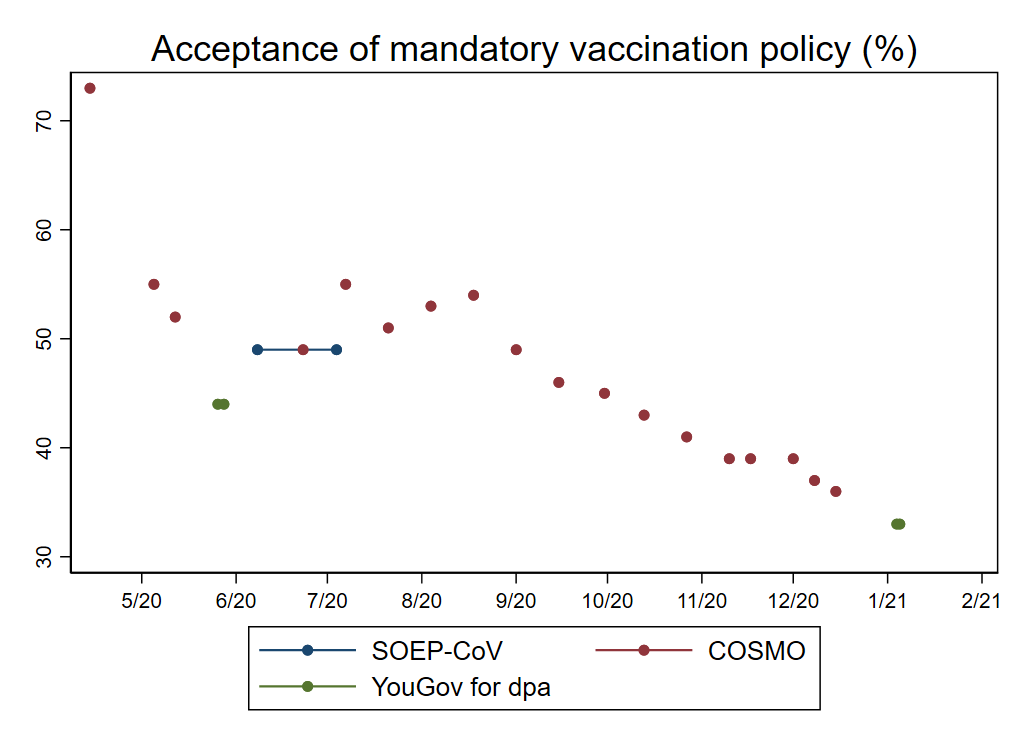


*Note*: Lines indicate the survey periods. If there are no lines, surveys were performed on a single day.
