# Supplementary material for: Attitudes on voluntary and mandatory vaccination against COVID-19: Evidence from Germany
Source: PLoS One. 2021 May 10;16(5):e0248372. doi: 10.1371/journal.pone.0248372 (PMC8109805; doi:10.1371/journal.pone.0248372)
Supplement: S3 File — (DOCX) [file pone.0248372.s003.docx]

# S3 File: Complementary Estimation Results

S3.1 Table provides statistical comparisons of the means across groups assuming equal variances across groups.

**S3.1 Table:** Comparisons of groups by arguments

|  | **P-values for group comparisons** | | | | | |  |
| --- | --- | --- | --- | --- | --- | --- | --- |
| **Argument** | 1 & 2 | 1 & 3 | 1 & 4 | 2 & 3 | 2 & 4 | 3 & 4 |  |
| Others’ willingness to get vaccinated without mandatory vaccination | 0.006 | 0.001 | 0.000 | 0.227 | 0.002 | 0.341 | |
| Misperception of risks | 0.013 | 0.001 | 0.000 | 0.000 | 0.000 | 0.556 | |
| Legitimacy of a policy of mandatory vaccinations in general | 0.845 | 0.062 | 0.000 | 0.033 | 0.000 | 0.425 | |
| Other reasons | 0.019 | 0.001 | 0.000 | 0.138 | 0.075 | 0.743 | |

*Note.* Data from SOEP and SOEP-CoV. P-values stem from t-tests of groupwise mean comparisons in Table 2. Results are weighted.

**S3.2 Table:** Comparison of characteristics by groups

|  | **P-values for group comparisons** | | | | | |
| --- | --- | --- | --- | --- | --- | --- |
| **Characteristics** | 1 & 2 | 1 & 3 | 1 & 4 | 2 & 3 | 2 & 4 | 3 & 4 |
| Female | 0.013 | 0.517 | 0.005 | 0.009 | 0.850 | 0.005 |
| Age | 0.543 | 0.122 | 0.001 | 0.214 | 0.002 | 0.711 |
| Tertiary Education | 0.000 | 0.999 | 0.000 | 0.001 | 0.669 | 0.002 |
| Net monthly income per household, 1k€ | 0.215 | 0.114 | 0.219 | 0.028 | 0.515 | 0.014 |
| Children younger than 16 | 0.561 | 0.624 | 0.868 | 0.925 | 0.608 | 0.678 |
| Eastern Federal States | 0.596 | 0.740 | 0.198 | 0.487 | 0.042 | 0.628 |
| Extraversion | 0.006 | 0.377 | 0.083 | 0.496 | 0.162 | 0.996 |
| Conscientiousness | 0.997 | 0.363 | 0.571 | 0.338 | 0.506 | 0.532 |
| Openness to experience | 0.012 | 0.141 | 0.038 | 0.780 | 0.355 | 0.806 |
| Neuroticism | 0.824 | 0.177 | 0.061 | 0.193 | 0.042 | 0.928 |
| Agreeableness | 0.233 | 0.021 | 0.090 | 0.093 | 0.540 | 0.184 |
| Willingness to take risks | 0.939 | 0.210 | 0.996 | 0.184 | 0.905 | 0.155 |
| Health: Self-assessment | 0.311 | 0.080 | 0.297 | 0.233 | 0.944 | 0.206 |
| Number of risky diseases | 0.169 | 0.008 | 0.001 | 0.054 | 0.129 | 0.226 |
| Test for COVID-19 in household | 0.440 | 0.282 | 0.819 | 0.142 | 0.499 | 0.223 |
| Positive test for COVID-19 in household | 0.228 | 0.228 | 0.228 | . | . | . |
| Prob. of life-threatening disease | 0.053 | 0.059 | 0.156 | 0.497 | 0.467 | 0.268 |
| Political preferences | 0.081 | 0.211 | 0.194 | 0.982 | 0.495 | 0.676 |

*Note.* Data from SOEP and SOEP-CoV. P-values stem from t-tests of groupwise mean comparisons in Table 3. Results are weighted.

Below, we relax the assumption of equal variances across groups as a robustness check. First, S3.3 Table displays tests for equal variances across groups. Only 10.78% (=11/102) of the tests reject the null hypothesis of equal variances across groups on a 5% level of significance. The same number is 14.71% (=15/102) for the 10% level of significance.

**S3.3 Table:** Comparison of variances of characteristics across groups

|  | **P-values for group comparisons** | | | | | |
| --- | --- | --- | --- | --- | --- | --- |
| **Characteristics** | 1 & 2 | 1 & 3 | 1 & 4 | 2 & 3 | 2 & 4 | 3 & 4 |
| Female | 0.584 | 0.573 | 0.430 | 0.772 | 0.849 | 0.839 |
| Age | 0.857 | 0.092* | 0.095* | 0.059* | 0.029** | 0.485 |
| Tertiary Education | 0.000*** | 0.999 | 0.001*** | 0.005*** | 0.664 | 0.008*** |
| Net monthly income per household, 1k€ | 0.243 | 0.326 | 0.036** | 0.292 | 0.319 | 0.599 |
| Children younger than 16 | 0.555 | 0.639 | 0.866 | 0.926 | 0.612 | 0.694 |
| Eastern Federal States | 0.594 | 0.733 | 0.216 | 0.460 | 0.048** | 0.646 |
| Extraversion | 0.446 | 0.603 | 0.371 | 0.902 | 0.947 | 0.928 |
| Conscientiousness | 0.505 | 0.831 | 0.649 | 0.136 | 0.580 | 0.245 |
| Openess to experience | 0.721 | 0.665 | 0.445 | 0.368 | 0.604 | 0.155 |
| Neuroticism | 0.835 | 0.893 | 0.957 | 0.988 | 0.742 | 0.849 |
| Agreeableness | 0.489 | 0.900 | 0.528 | 0.657 | 0.099* | 0.490 |
| Willingness to take risks | 0.112 | 0.402 | 0.122 | 0.013** | 0.910 | 0.014** |
| Health: Self-assessment | 0.688 | 0.364 | 0.962 | 0.502 | 0.622 | 0.336 |
| Number of risky diseases | 0.119 | 0.018** | 0.011** | 0.245 | 0.676 | 0.340 |
| Test for COVID-19 in household | 0.436 | 0.248 | 0.819 | 0.102 | 0.499 | 0.181 |
| Positive test for COVID-19 in household |  |  |  |  |  |  |
| Prob. of life-threatening disease | 0.643 | 0.375 | 0.138 | 0.582 | 0.237 | 0.673 |
| Political preferences | 0.664 | 0.215 | 0.730 | 0.334 | 0.877 | 0.264 |

*Note.* Data from SOEP and SOEP-CoV. P-values stem from $Chi^{2}$-tests of groupwise variance comparisons. Results for “Positive test for COVID-19 in household” are not available since there exists no positive test result for observations in group 2 to 4. Results are weighted. * p < 0.10, ** p < 0.05, *** p < 0.01.

Second, we performed group comparisons, relaxing the assumption of equal variances across groups. The results are displayed in S3.4 Table. The main takeaway is that our conclusions remain unchanged.

**S3.4 Table:** P-values for mean comparison across groups, relaxing the assumption of equal variances across groups

|  | **P-values for group comparisons** | | | | | |
| --- | --- | --- | --- | --- | --- | --- |
| **Characteristics** | 1 & 2 | 1 & 3 | 1 & 4 | 2 & 3 | 2 & 4 | 3 & 4 |
| Female | 0.013** | 0.519 | 0.005*** | 0.009*** | 0.850 | 0.005*** |
| Age | 0.544 | 0.125 | 0.001*** | 0.216 | 0.002*** | 0.711 |
| Tertiary Education | 0.000*** | 0.999 | 0.000*** | 0.001*** | 0.670 | 0.002*** |
| Net monthly income per household, 1k€ | 0.216 | 0.116 | 0.220 | 0.029** | 0.515 | 0.014** |
| Children younger than 16 | 0.561 | 0.625 | 0.868 | 0.926 | 0.608 | 0.679 |
| Eastern Federal States | 0.597 | 0.741 | 0.199 | 0.488 | 0.042** | 0.629 |
| Extraversion | 0.006*** | 0.380 | 0.084* | 0.498 | 0.162 | 0.996 |
| Conscientiousness | 0.997 | 0.366 | 0.572 | 0.341 | 0.507 | 0.534 |
| Openess to experience | 0.013** | 0.144 | 0.038** | 0.781 | 0.356 | 0.806 |
| Neuroticism | 0.824 | 0.180 | 0.061* | 0.195 | 0.042** | 0.929 |
| Agreeableness | 0.234 | 0.022** | 0.090* | 0.094* | 0.540 | 0.186 |
| Willingness to take risks | 0.939 | 0.213 | 0.996 | 0.186 | 0.905 | 0.157 |
| Health: Self-assessment | 0.312 | 0.082* | 0.298 | 0.235 | 0.944 | 0.207 |
| Number of risky diseases | 0.170 | 0.008*** | 0.001*** | 0.055* | 0.130 | 0.228 |
| Test for COVID-19 in household | 0.441 | 0.285 | 0.820 | 0.143 | 0.499 | 0.224 |
| Positive test for COVID-19 in household |  |  |  |  |  |  |
| Prob. of life-threatening disease | 0.054* | 0.061* | 0.157 | 0.499 | 0.468 | 0.269 |
| Political preferences | 0.082* | 0.214 | 0.195 | 0.983 | 0.495 | 0.677 |

*Note.* Data from SOEP and SOEP-CoV. P-values stem from t-tests of groupwise mean comparisons in Table 3. Results are weighted. * p < 0.10, ** p < 0.05, *** p < 0.01.
