# Supplementary material for: Attitudes on voluntary and mandatory vaccination against COVID-19: Evidence from Germany
Source: PLoS One. 2021 May 10;16(5):e0248372. doi: 10.1371/journal.pone.0248372 (PMC8109805; doi:10.1371/journal.pone.0248372)
Supplement: S4 File — (DOCX) [file pone.0248372.s004.docx]

# S4 File: Additional Diagnostics for the Logit Models

Table 4 in the main body of the paper provides initial model diagnostics in the form of the Pseudo-$R^{2}$, which is also referred to as McFadden’s $R^{2}$. This section provides two additional diagnostic tools. The first is the linktest. The idea behind the linktest is as follows: The logit estimations assume that the logarithm of the odds ratio is a linear function of the covariates and all relevant predictors are observed. If our model is correctly specified, we should not be able to detect any other predictors except by chance. To test this assumption, we calculate the linear combination of the covariates and the associated coefficient estimates of the respective logit estimation. We then regress our outcome on this linear prediction and this linear prediction squared. If correctly specified, we would expect that first, the linear combination of the coefficient estimates and the covariates should be significantly related to the outcome, and second, the squared term of this linear combination should not be significantly related to the respective outcome.

The results are summarized in S4.1 Table. The results indicate that our model is correctly specified. The linear combination of the covariates and the coefficient estimates are significantly associated with our outcomes in both panels, but their squares are not.

**S4.1 Table:** Linktest for the logit models for willingness to get vaccinated and attitudes towards obligatory vaccinations.

| Explanatory variable | Effect | S.E. | LB 95% CI | UB 95% CI | z-statistic | p-value |
| --- | --- | --- | --- | --- | --- | --- |
| Panel A: Willingness to get vaccinated |  |  |  |  |  |  |
| $x\hat{b}$ | 0.945 | 0.227 | 0.500 | 1.390 | 4.158 | 0.000 |
| $\left( x\hat{b} \right)^{2}$ | 0.032 | 0.111 | -0.186 | 0.249 | 0.284 | 0.776 |
| Panel B: Attitude toward mandatory vaccination |  |  |  |  |  |  |
| $x\hat{b}$ | 1.044 | 0.134 | 0.781 | 1.307 | 7.776 | 0.000 |
| $\left( x\hat{b} \right)^{2}$ | 0.145 | 0.144 | -0.137 | 0.427 | 1.007 | 0.314 |

*Note.* Data from SOEP and SOEP-CoV. All numbers unweighted. 2.1 Table displays the results for logit regressions of the indicator for individuals’ willingness to vaccinate and attitudes towards obligatory vaccinations on the linear combinations of the coefficient estimates and the associated covariates from the respective logit regressions underlying Tables 4 and 5.

An additional specification diagnostic includes the ROC analysis. The idea is that for each classification criterion, based on the prediction from the logit regressions, we can calculate the specificity and sensitivity of the criterion. A visual depiction of all combinations is the ROC curve. The ROC curve for the models in Tables 4 and 5 are depicted in S4.1 and S4.2 Figs, together with the estimate of the area under the curve. The area under the curve provides us with information on the accuracy of the predictions. In general, values in the range between 0.7 and 0.8 are considered acceptable [44, 45]. For the logit model explaining the individuals’ willingness to get vaccinated, the area under the curve is equal to 0.72. For the model explaining individuals’ attitudes towards obligatory vaccination is the area is 0.69. Thus, we conclude that the accuracy of our models is acceptable.

**S4.1 Fig**: Receiver operator curve of the Logit model for the willingness to get vaccinated


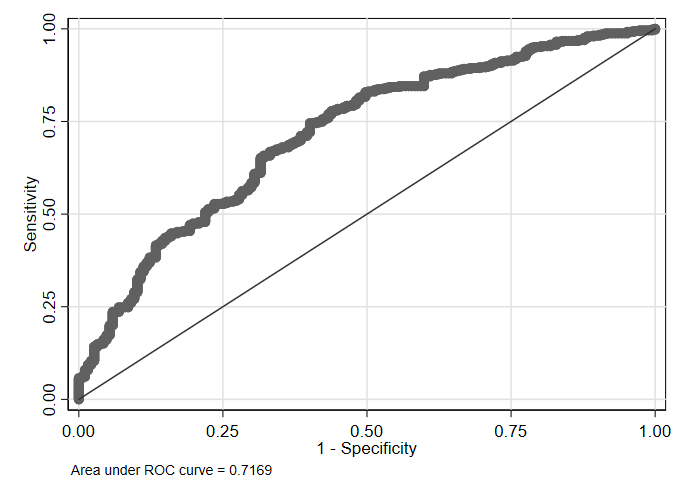


*Note.* Data from SOEP and SOEP-CoV. Fig 1 displays each combination of 1-specificity (false positive rate) and sensitivity (true positive rate) for each potential cut-off for predictions based on the model in Table 4.

**S4.2 Fig**: Receiver operator curve of the Logit model for attitudes toward mandatory vaccinations
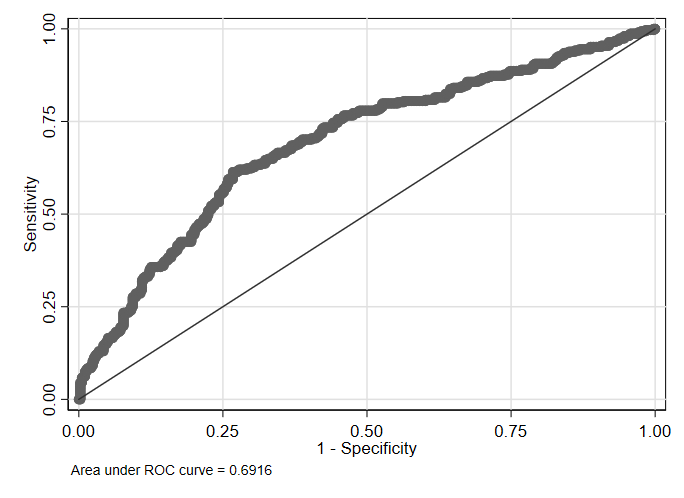


*Note.* Data from SOEP and SOEP-CoV. Fig S2.2 displays each combination of 1-specificity (false positive rate) and sensitivity (true positive rate) for each potential cut-off for predictions based on the model in Table 5.

[44] Mandrekar J. Receiver Operating Characteristic Curve in Diagnostic Test Assessment. Journal of Thoracic Oncology. 2020; (5)9: 1315-1316. https://doi.org/10.1097/JTO.0b013e3181ec173d.

[45] Stine R. Graphical Interpretation of Variance Inflation Factors. The American Statistician. 1995; 49(1): 53-56. http://doi.org/10.1080/00031305.1995.10476113.
