# Supplementary material for: Attitudes on voluntary and mandatory vaccination against COVID-19: Evidence from Germany
Source: PLoS One. 2021 May 10;16(5):e0248372. doi: 10.1371/journal.pone.0248372 (PMC8109805; doi:10.1371/journal.pone.0248372)
Supplement: S5 File — (DOCX) [file pone.0248372.s005.docx]

## S5 File: Multicollinearity Across Explanatory Variables

To assess the degree of multicollinearity across explanatory variables, we calculate the variance inflation factor for each of the covariates of our two logit models. The results are displayed in S5.1 and S5.2 Figs. Vertical bars display the variance inflation factor for each variable separately. There is no consensus on critical values for variance inflation factors. Some authors deem variance inflation factors above ten as problematic. Others suggest that variance inflation factors above five are problematic. In S5.1 and S5.2 Figs, we observe that age has a variance inflation factor of 7.6 and 7.7, respectively. In general, we consider age an important explanatory variable for the willingness to get vaccinated from a conceptual perspective. However, we investigate the model stability in Tables S5.1 and S5.2. Clearly, the results remain unchanged if we drop age from our logit models. In conclusion, we find that multicollinearity is not a concern in our estimations.

**S5.1 Fig**: Variance inflation factor of the covariates in the logit model explaining the willingness to get vaccinated


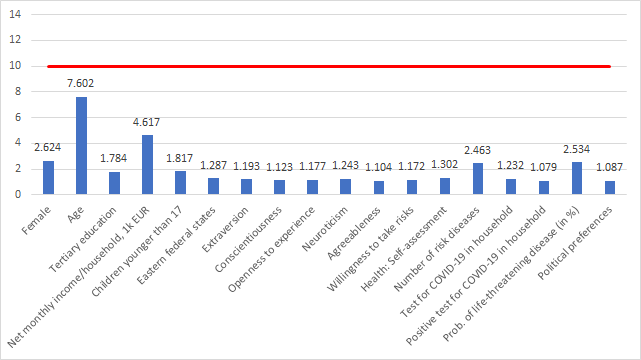


*Note.* Data from SOEP and SOEP-CoV. S3.1 Fig displays the variance inflation factor for each of the covariates in the Logit estimation displayed in Table 4. The vertical bar indicates variance inflation factors equal to ten.

**S5.2 Fig**: Variance inflation factor of the covariates in the logit model for attitudes toward mandatory vaccinations


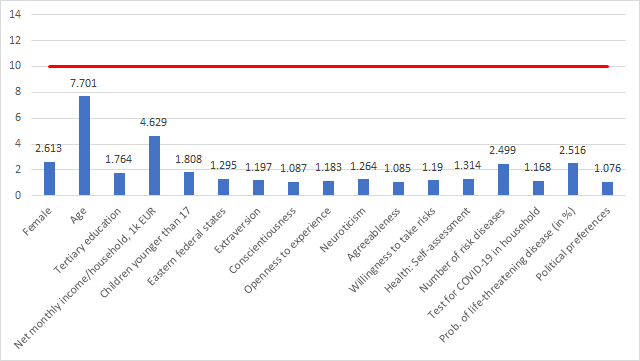


*Note.* Data from SOEP and SOEP-CoV. S3.2 Fig displays the variance inflation factor for each of the covariates in the Logit estimation displayed in Table 5. The vertical bar indicates variance inflation factors equal to ten.

**S5.1 Table:** Average marginal effects of individual characteristics on willingness to get vaccinated, excluding age (N=678, Pseudo R2 of underlying Logit estimation: 0.094)

| Explanatory variable | Effect | S.E. | LB 95% CI | UB 95% CI | z-statistic | p-value |
| --- | --- | --- | --- | --- | --- | --- |
| Female | -0.097 | 0.036 | -0.167 | -0.027 | -2.717 | 0.007 |
| Tertiary education | 0.137 | 0.036 | 0.067 | 0.208 | 3.837 | 0.000 |
| Net monthly income per household, 1k EUR | 0.025 | 0.012 | 0.001 | 0.049 | 2.034 | 0.042 |
| Children younger than 17 | -0.040 | 0.039 | -0.116 | 0.037 | -1.014 | 0.311 |
| Eastern federal states | 0.011 | 0.039 | -0.067 | 0.088 | 0.272 | 0.786 |
| Extraversion | -0.010 | 0.018 | -0.045 | 0.025 | -0.549 | 0.583 |
| Conscientiousness | -0.018 | 0.018 | -0.054 | 0.017 | -1.018 | 0.309 |
| Openness to experience | 0.027 | 0.018 | -0.009 | 0.063 | 1.482 | 0.138 |
| Neuroticism | -0.032 | 0.019 | -0.069 | 0.005 | -1.670 | 0.095 |
| Agreeableness | -0.016 | 0.018 | -0.051 | 0.019 | -0.886 | 0.376 |
| Willingness to take risks | -0.031 | 0.018 | -0.067 | 0.005 | -1.706 | 0.088 |

| Health: Self-assessment | -0.003 | 0.019 | -0.040 | 0.034 | -0.155 | 0.877 |
| --- | --- | --- | --- | --- | --- | --- |
| Number of risk diseases | 0.036 | 0.018 | 0.000 | 0.072 | 1.935 | 0.053 |
| Test for COVID-19 in household | -0.063 | 0.053 | -0.166 | 0.040 | -1.195 | 0.232 |
| Positive test for COVID-19 in household | -0.312 | 0.302 | -0.904 | 0.279 | -1.035 | 0.301 |
| Prob. of life-threatening disease (in %) | 0.003 | 0.001 | 0.001 | 0.005 | 3.617 | 0.000 |
| Political preferences | 0.012 | 0.016 | -0.020 | 0.044 | 0.740 | 0.459 |

*Note.* Data from SOEP and SOEP-CoV. All numbers unweighted. Column “Explanatory variable” indicates data surveyed in years different from year 2020. S.E. denotes standard error. LB denotes lower and UB upper bound of the confidence band (CI). S1.1 Table in the S1 File provides definitions of all the variables. Marginal effects. The Big Five, risk taking, self-assessed health and political orientation are measured in standard deviations. For political preferences, higher values are associated with a left political orientation.

**S5.2 Table:** Average marginal effects of individual characteristics on attitudes toward mandatory vaccinations, excluding age (N=682, Pseudo R2 of underlying Logit estimation: 0.063)

| Explanatory variable | Effect | S.E. | LB 95% CI | UB 95% CI | z-stat. | p-value |
| --- | --- | --- | --- | --- | --- | --- |
| Female | -0.089 | 0.039 | -0.165 | -0.014 | -2.310 | 0.021 |
| Tertiary education | -0.036 | 0.042 | -0.119 | 0.047 | -0.851 | 0.395 |
| Net monthly income per household, 1k EUR | 0.005 | 0.011 | -0.016 | 0.026 | 0.455 | 0.649 |
| Children younger than 17 | -0.028 | 0.044 | -0.113 | 0.057 | -0.645 | 0.519 |
| Eastern federal states | 0.158 | 0.044 | 0.071 | 0.245 | 3.552 | 0.000 |
| Extraversion | 0.002 | 0.019 | -0.036 | 0.040 | 0.120 | 0.904 |
| Conscientiousness | 0.012 | 0.020 | -0.026 | 0.050 | 0.620 | 0.535 |
| Openness to experience | -0.005 | 0.020 | -0.044 | 0.033 | -0.277 | 0.782 |
| Neuroticism | -0.057 | 0.020 | -0.096 | -0.017 | -2.802 | 0.005 |
| Agreeableness | 0.007 | 0.019 | -0.032 | 0.045 | 0.336 | 0.737 |
| Willingness to take risks | 0.002 | 0.020 | -0.038 | 0.042 | 0.088 | 0.930 |
| Health: Self-assessment | 0.005 | 0.021 | -0.036 | 0.046 | 0.225 | 0.822 |
| Number of risk diseases | 0.051 | 0.020 | 0.013 | 0.089 | 2.611 | 0.009 |
| Test for COVID-19 in household | -0.011 | 0.059 | -0.125 | 0.104 | -0.183 | 0.855 |
| Positive test for COVID-19 in household | . | . | . | . | . | . |
| Prob. of life-threatening disease (in %) | 0.003 | 0.001 | 0.001 | 0.005 | 3.561 | 0.000 |
| Political preferences | 0.000 | 0.018 | -0.036 | 0.036 | 0.001 | 0.999 |

*Note.* Data from SOEP and SOEP-CoV. All numbers unweighted. Column “Explanatory variable” indicates data surveyed in years different from year 2020. S.E. denotes standard error. LB denotes lower and UB upper bound of the confidence band (CI). S1.1 Table in the S1 File provides definitions of all the variables. Marginal effects. The Big Five, risk taking, self-assessed health and political orientation are measured in standard deviations. For political preferences, higher values are associated with a left political orientation.
