# Supplementary material for: Attitudes on voluntary and mandatory vaccination against COVID-19: Evidence from Germany
Source: PLoS One. 2021 May 10;16(5):e0248372. doi: 10.1371/journal.pone.0248372 (PMC8109805; doi:10.1371/journal.pone.0248372)
Supplement: S6 File — (DOCX) [file pone.0248372.s006.docx]

# S6 File: Functional Form Assumptions and Simultaneity

So far, we assumed that the logit represents the exact functional form. Further, we assumed responses to the two items (attitudes to voluntary and mandatory vaccination) are independent. In this section, we first relax the first assumption and estimate probit models. Second, we relax the assumption of independent responses across both items. Therefore, in this section, we restricted the sample to respondents answering both items. Tables S6.1 to S6.4 display probit models and average marginal effects for our two dependent variables. Clearly, the average marginal effects indicate that the functional form does not matter much for the conclusions.

Further, Tables S6.5 and S6.6 display the results based on a multivariate probit in which errors are allowed to be correlated across models. Clearly, the cross-equation correlation of the errors is 0.507 and significantly different from zero (p-value=0.000, chi2(1) =56.663). However, comparing the results of the multivariate probits Tables S6.5 and S6.6 with the probit models, in which we enforce the cross-equation correlation of errors to be zero, in Tables S6.1 and S6.3 suggest that our qualitative results are not altered by relaxing this assumption.

**S6.1 Table:** Coefficient estimates of probit model of individual characteristics on willingness to get vaccinated (N= 653, Pseudo R2: 0.105)

| Explanatory variable | Effect | S.E. | LB 95% CI | UB 95% CI | z-statistic | p-value |
| --- | --- | --- | --- | --- | --- | --- |
| Female | -0.348 | 0.121 | -0.585 | -0.110 | -2.865 | 0.004 |
| Age | 0.012 | 0.004 | 0.004 | 0.021 | 2.809 | 0.005 |
| Tertiary education | 0.467 | 0.133 | 0.206 | 0.727 | 3.513 | 0.000 |
| Net monthly income per household, 1k EUR | 0.073 | 0.037 | 0.000 | 0.146 | 1.956 | 0.050 |
| Children younger than 17 | -0.025 | 0.136 | -0.291 | 0.241 | -0.185 | 0.853 |
| Eastern federal states | 0.024 | 0.138 | -0.246 | 0.294 | 0.175 | 0.861 |
| Extraversion | -0.056 | 0.059 | -0.171 | 0.060 | -0.942 | 0.346 |
| Conscientiousness | -0.064 | 0.061 | -0.184 | 0.056 | -1.050 | 0.294 |
| Openness to experience | 0.108 | 0.062 | -0.013 | 0.229 | 1.754 | 0.079 |
| Neuroticism | -0.096 | 0.063 | -0.220 | 0.028 | -1.511 | 0.131 |
| Agreeableness | -0.091 | 0.059 | -0.206 | 0.024 | -1.553 | 0.120 |
| Willingness to take risks | -0.065 | 0.062 | -0.186 | 0.056 | -1.052 | 0.293 |

| Health: Self-assessment | -0.009 | 0.062 | -0.130 | 0.112 | -0.147 | 0.883 |
| --- | --- | --- | --- | --- | --- | --- |
| Number of risk diseases | 0.036 | 0.063 | -0.088 | 0.161 | 0.575 | 0.565 |
| Test for COVID-19 in household | -0.148 | 0.164 | -0.469 | 0.173 | -0.901 | 0.368 |
| Positive test for COVID-19 in household | n.a. | n.a. | n.a. | n.a. | n.a. | n.a. |
| Prob. of life-threatening disease (in %) | 0.010 | 0.003 | 0.004 | 0.015 | 3.448 | 0.001 |
| Political preferences | 0.030 | 0.055 | -0.079 | 0.138 | 0.537 | 0.591 |
| Constant | -0.456 | 0.276 | -0.997 | 0.086 | -1.649 | 0.099 |

*Note.* Data from SOEP and SOEP-CoV. All numbers unweighted. Column “Explanatory variable” indicates data surveyed in years different from year 2020. S.E. denotes standard error. LB denotes lower and UB upper bound of the confidence band (CI). S1.1 Table in the S1 File provides definitions of all the variables. Marginal effects. The Big Five, risk taking, self-assessed health and political orientation are measured in standard deviations. For political preferences, higher values are associated with a left political orientation.

**S6.2 Table:** Average marginal effects for probit model of individual characteristics on willingness to get vaccinated (N= 653, Pseudo R2: 0.105)

| Explanatory variable | Effect | S.E. | LB 95% CI | UB 95% CI | z-statistic | p-value |
| --- | --- | --- | --- | --- | --- | --- |
| Female | -0.103 | 0.035 | -0.172 | -0.033 | -2.900 | 0.004 |
| Age | 0.004 | 0.001 | 0.001 | 0.006 | 2.866 | 0.004 |
| Tertiary education | 0.134 | 0.036 | 0.064 | 0.205 | 3.724 | 0.000 |
| Net monthly income per household, 1k EUR | 0.022 | 0.011 | 0.000 | 0.043 | 1.974 | 0.048 |
| Children younger than 17 | -0.007 | 0.041 | -0.087 | 0.072 | -0.185 | 0.854 |
| Eastern federal states | 0.007 | 0.041 | -0.073 | 0.087 | 0.176 | 0.860 |
| Extraversion | -0.017 | 0.018 | -0.051 | 0.018 | -0.943 | 0.346 |
| Conscientiousness | -0.019 | 0.018 | -0.055 | 0.016 | -1.052 | 0.293 |
| Openness to experience | 0.032 | 0.018 | -0.004 | 0.068 | 1.766 | 0.077 |
| Neuroticism | -0.028 | 0.019 | -0.065 | 0.008 | -1.516 | 0.130 |
| Agreeableness | -0.027 | 0.017 | -0.061 | 0.007 | -1.559 | 0.119 |
| Willingness to take risks | -0.019 | 0.018 | -0.055 | 0.016 | -1.056 | 0.291 |

| Health: Self-assessment | -0.003 | 0.018 | -0.039 | 0.033 | -0.147 | 0.883 |
| --- | --- | --- | --- | --- | --- | --- |
| Number of risk diseases | 0.011 | 0.019 | -0.026 | 0.048 | 0.576 | 0.565 |
| Test for COVID-19 in household | -0.045 | 0.051 | -0.146 | 0.056 | -0.877 | 0.380 |
| Positive test for COVID-19 in household | .n.a. | n.a.. | n.a.. | n.a.. | n.a. | n.a. |
| Prob. of life-threatening disease (in %) | 0.003 | 0.001 | 0.001 | 0.005 | 3.537 | 0.000 |
| Political preferences | 0.009 | 0.016 | -0.023 | 0.041 | 0.538 | 0.590 |

*Note.* Data from SOEP and SOEP-CoV. All numbers unweighted. Column “Explanatory variable” indicates data surveyed in years different from year 2020. S.E. denotes standard error. LB denotes lower and UB upper bound of the confidence band (CI). S1.1 Table in the S1 File provides definitions of all the variables. Marginal effects. The Big Five, risk taking, self-assessed health and political orientation are measured in standard deviations. For political preferences, higher values are associated with a left political orientation.

**S6.3 Table:** Coefficient estimates of probit model of individual characteristics on attitudes toward mandatory vaccination (N= 653, Pseudo R2: 0.084)

| Explanatory variable | Effect | S.E. | LB 95% CI | UB 95% CI | z-statistic | p-value |
| --- | --- | --- | --- | --- | --- | --- |
| Female | -0.225 | 0.110 | -0.442 | -0.009 | -2.039 | 0.041 |
| Age | 0.017 | 0.004 | 0.008 | 0.025 | 3.971 | 0.000 |
| Tertiary education | -0.112 | 0.119 | -0.345 | 0.120 | -0.946 | 0.344 |
| Net monthly income per household, 1k EUR | 0.017 | 0.031 | -0.043 | 0.076 | 0.543 | 0.587 |
| Children younger than 17 | 0.107 | 0.131 | -0.150 | 0.365 | 0.816 | 0.414 |
| Eastern federal states | 0.412 | 0.128 | 0.162 | 0.663 | 3.227 | 0.001 |
| Extraversion | -0.004 | 0.054 | -0.110 | 0.102 | -0.076 | 0.939 |
| Conscientiousness | 0.018 | 0.055 | -0.090 | 0.127 | 0.332 | 0.740 |
| Openness to experience | 0.001 | 0.055 | -0.107 | 0.108 | 0.015 | 0.988 |
| Neuroticism | -0.119 | 0.057 | -0.232 | -0.007 | -2.079 | 0.038 |
| Agreeableness | 0.007 | 0.054 | -0.099 | 0.113 | 0.130 | 0.896 |
| Willingness to take risks | 0.027 | 0.057 | -0.084 | 0.138 | 0.481 | 0.631 |
| Health: Self-assessment | -0.012 | 0.058 | -0.125 | 0.101 | -0.207 | 0.836 |
| Number of risk diseases | 0.073 | 0.058 | -0.041 | 0.187 | 1.247 | 0.212 |
| Test for COVID-19 in household | 0.033 | 0.163 | -0.286 | 0.352 | 0.204 | 0.839 |
| Positive test for COVID-19 in household | n.a. | n.a. | n.a. | n.a. | n.a. | n.a. |
| Prob. of life-threatening disease (in %) | 0.008 | 0.003 | 0.003 | 0.013 | 3.082 | 0.002 |
| Political preferences | -0.027 | 0.052 | -0.129 | 0.075 | -0.521 | 0.602 |
| Constant | -1.281 | 0.272 | -1.815 | -0.747 | -4.701 | 0.000 |

*Note.* Data from SOEP and SOEP-CoV. All numbers unweighted. Column “Explanatory variable” indicates data surveyed in years different from year 2020. S.E. denotes standard error. LB denotes lower and UB upper bound of the confidence band (CI). S1.1 Table in the S1 File provides definitions of all the variables. Marginal effects. The Big Five, risk taking, self-assessed health and political orientation are measured in standard deviations. For political preferences, higher values are associated with a left political orientation.

**S6.4 Table:** Average marginal effects for probit model of individual characteristics on attitudes toward mandatory vaccinations (N= 653, Pseudo R2: 0.084)

| Explanatory variable | Effect | S.E. | LB 95% CI | UB 95% CI | z-statistic | p-value |
| --- | --- | --- | --- | --- | --- | --- |
| Female | -0.082 | 0.040 | -0.161 | -0.003 | -2.035 | 0.042 |
| Age | 0.006 | 0.001 | 0.003 | 0.009 | 4.132 | 0.000 |
| Tertiary education | -0.041 | 0.043 | -0.125 | 0.043 | -0.949 | 0.343 |
| Net monthly income per household, 1k EUR | 0.006 | 0.011 | -0.016 | 0.028 | 0.544 | 0.587 |
| Children younger than 17 | 0.039 | 0.047 | -0.053 | 0.130 | 0.822 | 0.411 |
| Eastern federal states | 0.151 | 0.046 | 0.060 | 0.242 | 3.265 | 0.001 |
| Extraversion | -0.001 | 0.020 | -0.040 | 0.037 | -0.076 | 0.939 |
| Conscientiousness | 0.007 | 0.020 | -0.033 | 0.046 | 0.333 | 0.739 |
| Openness to experience | 0.000 | 0.020 | -0.039 | 0.039 | 0.015 | 0.988 |
| Neuroticism | -0.043 | 0.021 | -0.083 | -0.003 | -2.101 | 0.036 |
| Agreeableness | 0.003 | 0.020 | -0.036 | 0.041 | 0.130 | 0.896 |
| Willingness to take risks | 0.010 | 0.020 | -0.030 | 0.050 | 0.481 | 0.631 |
| Health: Self-assessment | -0.004 | 0.021 | -0.045 | 0.037 | -0.207 | 0.836 |
| Number of risk diseases | 0.026 | 0.021 | -0.015 | 0.067 | 1.253 | 0.210 |
| Test for COVID-19 in household | 0.012 | 0.059 | -0.104 | 0.128 | 0.203 | 0.839 |
| Positive test for COVID-19 in household | .n.a. | n.a.. | n.a.. | n.a.. | n.a. | n.a. |
| Prob. of life-threatening disease (in %) | 0.003 | 0.001 | 0.001 | 0.005 | 3.157 | 0.002 |
| Political preferences | -0.010 | 0.019 | -0.047 | 0.027 | -0.522 | 0.602 |

*Note.* Data from SOEP and SOEP-CoV. All numbers unweighted. Column “Explanatory variable” indicates data surveyed in years different from year 2020. S.E. denotes standard error. LB denotes lower and UB upper bound of the confidence band (CI). S1.1 Table in the S1 File provides definitions of all the variables. Marginal effects. The Big Five, risk taking, self-assessed health and political orientation are measured in standard deviations. For political preferences, higher values are associated with a left political orientation.

**S6.5 Table:** Results for multivariate probit model of individual characteristics on willingness to get vaccinated (N= 653)

| Explanatory variable | Effect | S.E. | LB 95% CI | UB 95% CI | z-statistic | p-value |
| --- | --- | --- | --- | --- | --- | --- |
| Female | -0.359 | 0.119 | -0.592 | -0.126 | -3.018 | 0.003 |
| Age | 0.012 | 0.004 | 0.003 | 0.021 | 2.675 | 0.007 |
| Tertiary education | 0.428 | 0.129 | 0.175 | 0.682 | 3.313 | 0.001 |
| Net monthly income per household, 1k EUR | 0.074 | 0.036 | 0.004 | 0.145 | 2.066 | 0.039 |
| Children younger than 17 | -0.020 | 0.134 | -0.283 | 0.243 | -0.149 | 0.882 |
| Eastern federal states | 0.058 | 0.141 | -0.218 | 0.333 | 0.409 | 0.682 |
| Extraversion | -0.062 | 0.058 | -0.175 | 0.051 | -1.072 | 0.284 |
| Conscientiousness | -0.057 | 0.062 | -0.178 | 0.064 | -0.923 | 0.356 |
| Openness to experience | 0.107 | 0.060 | -0.010 | 0.225 | 1.790 | 0.073 |
| Neuroticism | -0.090 | 0.062 | -0.212 | 0.032 | -1.441 | 0.150 |
| Agreeableness | -0.105 | 0.059 | -0.220 | 0.010 | -1.788 | 0.074 |
| Willingness to take risks | -0.054 | 0.061 | -0.173 | 0.066 | -0.879 | 0.379 |
| Health: Self-assessment | -0.025 | 0.061 | -0.145 | 0.094 | -0.414 | 0.679 |
| Number of risk diseases | 0.031 | 0.062 | -0.091 | 0.153 | 0.495 | 0.620 |
| Test for COVID-19 in household | -0.161 | 0.166 | -0.486 | 0.164 | -0.969 | 0.333 |
| Positive test for COVID-19 in household | 0.009 | 0.003 | 0.004 | 0.015 | 3.374 | 0.001 |
| Prob. of life-threatening disease (in %) | 0.018 | 0.054 | -0.088 | 0.125 | 0.335 | 0.737 |
| Political preferences | -0.427 | 0.277 | -0.970 | 0.117 | -1.538 | 0.124 |

*Note.* Data from SOEP and SOEP-CoV. All numbers unweighted. Column “Explanatory variable” indicates data surveyed in years different from year 2020. S.E. denotes standard error. LB denotes lower and UB upper bound of the confidence band (CI). S1.1 Table in the S1 File provides definitions of all the variables. Marginal effects. The Big Five, risk taking, self-assessed health and political orientation are measured in standard deviations. For political preferences, higher values are associated with a left political orientation.

**S6.6 Table:** Results for multivariate probit estimation of individual characteristics on support for mandatory vaccination (N= 653)

| Explanatory variable | Effect | S.E. | LB 95% CI | UB 95% CI | z-statistic | p-value |
| --- | --- | --- | --- | --- | --- | --- |
| Female | -0.226 | 0.110 | -0.442 | -0.010 | -2.050 | 0.040 |
| Age | 0.017 | 0.004 | 0.008 | 0.025 | 3.957 | 0.000 |
| Tertiary education | -0.108 | 0.117 | -0.338 | 0.122 | -0.921 | 0.357 |
| Net monthly income per household, 1k EUR | 0.017 | 0.031 | -0.044 | 0.078 | 0.543 | 0.587 |
| Children younger than 17 | 0.099 | 0.130 | -0.157 | 0.354 | 0.756 | 0.450 |
| Eastern federal states | 0.419 | 0.127 | 0.169 | 0.669 | 3.285 | 0.001 |
| Extraversion | 0.000 | 0.055 | -0.108 | 0.107 | -0.005 | 0.996 |
| Conscientiousness | 0.018 | 0.056 | -0.092 | 0.127 | 0.314 | 0.753 |
| Openness to experience | -0.010 | 0.054 | -0.116 | 0.097 | -0.175 | 0.861 |
| Neuroticism | -0.126 | 0.057 | -0.238 | -0.014 | -2.202 | 0.028 |
| Agreeableness | 0.015 | 0.054 | -0.092 | 0.121 | 0.268 | 0.788 |
| Willingness to take risks | 0.029 | 0.055 | -0.079 | 0.136 | 0.527 | 0.598 |
| Health: Self-assessment | -0.010 | 0.058 | -0.124 | 0.103 | -0.179 | 0.858 |
| Number of risk diseases | 0.069 | 0.057 | -0.043 | 0.181 | 1.207 | 0.227 |
| Test for COVID-19 in household | 0.011 | 0.165 | -0.312 | 0.335 | 0.069 | 0.945 |
| Positive test for COVID-19 in household | 0.008 | 0.003 | 0.003 | 0.013 | 3.202 | 0.001 |
| Prob. of life-threatening disease (in %) | -0.029 | 0.052 | -0.131 | 0.074 | -0.552 | 0.581 |
| Political preferences | -0.226 | 0.110 | -0.442 | -0.010 | -2.050 | 0.040 |

*Note.* Data from SOEP and SOEP-CoV. All numbers unweighted. Column “Explanatory variable” indicates data surveyed in years different from year 2020. S.E. denotes standard error. LB denotes lower and UB upper bound of the confidence band (CI). S1.1 Table in the S1 File provides definitions of all the variables. Marginal effects. The Big Five, risk taking, self-assessed health and political orientation are measured in standard deviations. For political preferences, higher values are associated with a left political orientation.
