# Supplementary material for: Attitudes on voluntary and mandatory vaccination against COVID-19: Evidence from Germany
Source: PLoS One. 2021 May 10;16(5):e0248372. doi: 10.1371/journal.pone.0248372 (PMC8109805; doi:10.1371/journal.pone.0248372)
Supplement: S7 File — (DOCX) [file pone.0248372.s007.docx]

## S7 File: Imputation

It is possible that respondents who did not give an answer about their vaccination preferences – for example, because they are still undecided – would decide to vaccinate or support mandatory vaccination after an adequate vaccination campaign. In a robustness check, we followed this argument by assigning respondents who refused to answer the question about voluntary or mandatory vaccination to the ‘yes’ category and repeated the logit estimation. This does not change our conclusions results (see S7.1 and S7.2 Tables).

**S7.1 Table:** Average marginal effects of individual characteristics on willingness to get vaccinated (N= 710, Pseudo R2 of underlying Logit estimation: 0.094)

| Explanatory variable | Effect | S.E. | LB 95% CI | UB 95% CI | z-statistic | p-value |
| --- | --- | --- | --- | --- | --- | --- |
| Female | -0.084 | 0.034 | -0.151 | -0.017 | -2.460 | 0.014 |
| Age | 0.003 | 0.001 | 0.001 | 0.006 | 2.710 | 0.007 |
| Tertiary education | 0.118 | 0.035 | 0.050 | 0.186 | 3.395 | 0.001 |
| Net monthly income per household, 1k EUR | 0.022 | 0.012 | -0.001 | 0.046 | 1.890 | 0.059 |
| Children younger than 17 | -0.007 | 0.038 | -0.082 | 0.068 | -0.185 | 0.853 |
| Eastern federal states | 0.002 | 0.039 | -0.074 | 0.078 | 0.040 | 0.968 |
| Extraversion | -0.010 | 0.017 | -0.043 | 0.023 | -0.601 | 0.548 |
| Conscientiousness | -0.020 | 0.018 | -0.055 | 0.015 | -1.129 | 0.259 |
| Openness to experience | 0.030 | 0.018 | -0.006 | 0.065 | 1.652 | 0.098 |
| Neuroticism | -0.021 | 0.019 | -0.057 | 0.016 | -1.118 | 0.263 |
| Agreeableness | -0.021 | 0.017 | -0.055 | 0.012 | -1.258 | 0.208 |
| Willingness to take risks | -0.024 | 0.018 | -0.059 | 0.010 | -1.375 | 0.169 |
| Health: Self-assessment | -0.002 | 0.018 | -0.038 | 0.033 | -0.137 | 0.891 |
| Number of risk diseases | 0.017 | 0.019 | -0.020 | 0.053 | 0.909 | 0.363 |
| Test for COVID-19 in household | -0.045 | 0.049 | -0.141 | 0.050 | -0.933 | 0.351 |
| Positive test for COVID-19 in household | -0.344 | 0.307 | -0.945 | 0.257 | -1.122 | 0.262 |
| Prob. of life-threatening disease (in %) | 0.003 | 0.001 | 0.001 | 0.004 | 3.213 | 0.001 |
| Political preferences | 0.014 | 0.016 | -0.016 | 0.045 | 0.914 | 0.361 |

*Note.* Data from SOEP and SOEP-CoV. All numbers unweighted. Column “Explanatory variable” indicates data surveyed in years different from year 2020. S.E. denotes standard error. LB denotes lower and UB upper bound of the confidence band (CI). S1.1 Table in the S1 File provides definitions of all the variables. Marginal effects. The Big Five, risk taking, self-assessed health and political orientation are measured in standard deviations. For political preferences, higher values are associated with a left political orientation.

**S7.2 Table:** Average marginal effects of individual characteristics on attitudes toward mandatory vaccinations (N=710, Pseudo R2 of underlying Logit estimation: 0.074)

| Explanatory variable | Effect | S.E. | LB 95% CI | UB 95% CI | z-stat. | p-value |
| --- | --- | --- | --- | --- | --- | --- |
| Female | -0.080 | 0.039 | -0.156 | -0.003 | -2.038 | 0.042 |
| Age | 0.006 | 0.001 | 0.003 | 0.009 | 3.945 | 0.000 |
| Tertiary education | -0.053 | 0.042 | -0.134 | 0.029 | -1.270 | 0.204 |
| Net monthly income per household, 1k EUR | 0.003 | 0.011 | -0.018 | 0.024 | 0.261 | 0.794 |
| Children younger than 17 | 0.029 | 0.046 | -0.061 | 0.119 | 0.633 | 0.527 |
| Eastern federal states | 0.144 | 0.045 | 0.056 | 0.232 | 3.207 | 0.001 |
| Extraversion | 0.009 | 0.019 | -0.028 | 0.046 | 0.473 | 0.636 |
| Conscientiousness | 0.017 | 0.019 | -0.021 | 0.055 | 0.885 | 0.376 |
| Openness to experience | 0.001 | 0.019 | -0.037 | 0.038 | 0.043 | 0.966 |
| Neuroticism | -0.042 | 0.020 | -0.081 | -0.003 | -2.107 | 0.035 |
| Agreeableness | -0.005 | 0.019 | -0.041 | 0.032 | -0.246 | 0.806 |
| Willingness to take risks | -0.002 | 0.020 | -0.041 | 0.037 | -0.104 | 0.917 |
| Health: Self-assessment | -0.005 | 0.020 | -0.045 | 0.035 | -0.233 | 0.816 |
| Number of risk diseases | 0.021 | 0.021 | -0.019 | 0.062 | 1.035 | 0.301 |
| Test for COVID-19 in household | -0.009 | 0.057 | -0.121 | 0.104 | -0.157 | 0.875 |
| Positive test for COVID-19 in household | -0.081 | 0.275 | -0.620 | 0.459 | -0.293 | 0.770 |
| Prob. of life-threatening disease (in %) | 0.003 | 0.001 | 0.001 | 0.004 | 3.026 | 0.002 |
| Political preferences | 0.002 | 0.018 | -0.034 | 0.038 | 0.099 | 0.921 |

*Note.* Data from SOEP and SOEP-CoV. All numbers unweighted. Column “Explanatory variable” indicates data surveyed in years different from year 2020. S.E. denotes standard error. LB denotes lower and UB upper bound of the confidence band (CI). S1.1 Table in the S1 File provides definitions of all the variables. Marginal effects. The Big Five, risk taking, self-assessed health and political orientation are measured in standard deviations. For political preferences, higher values are associated with a left political orientation.
