# Supplementary material for: Attitudes on voluntary and mandatory vaccination against COVID-19: Evidence from Germany
Source: PLoS One. 2021 May 10;16(5):e0248372. doi: 10.1371/journal.pone.0248372 (PMC8109805; doi:10.1371/journal.pone.0248372)
Supplement: S8 File — (DOCX) [file pone.0248372.s008.docx]

# S8 File: Stata code

/*********************************************************************************/

/* program count_stars : return a string with the right number of stars */

/*********************************************************************************/

cap prog drop count_stars

prog def count_stars, rclass

{

syntax, p(real)

local star = ""

if `p' <= 0.1 local star = "*"

if `p' <= 0.05 local star = "**"

if `p' <= 0.01 local star = "***"

return local stars = "`star'"

}

end

* Folder structure

global Out "F:\Impfung\Out\"

global Data "F:\Impfung\data"

global Data_prep "F:\Impfung\data"

global tables "F:\Impfung\tables\"

global figures "F:\Impfung\figures\"

ssc install mvprobit

global covversion "20200805"

******************** DATA PREPARATION *************************

use "$Data\soep_cov_${covversion}_statav13.dta", clear

su pid if syear == 2020

di "We have `r(N)' observations in SOEP-CoV"

gen double p_severeill = .

replace p_severeill = pcovwkt1

replace p_severeill = 100 if pcovwkt2 == 1

gen byte d_severeill = .

replace d_severeill = 1 if pcovwkt2 == 1

replace d_severeill = 0 if pcovwkt2 == .b

* Prevalence, symptoms, Quarantaine etc.

rename pcovtest pcovtest1

rename pcovtestm pcovtest1m

rename pcovtestt pcovtest1t

rename pcovteste pcovtest1e

rename phqua phqua1

rename pgrippe pgrippe1

rename pcovtest2_l1 pcovtest13

rename pcovtest2m_l1 pcovtest13m

rename pcovtest2t_l1 pcovtest13t

rename pcovtest2e_l1 pcovtest13e

rename phqua2_l1 phqua13

rename pgrippe2_l1 pgrippe13

rename pcovtest2_l2 pcovtest14

rename pcovtest2m_l2 pcovtest14m

rename pcovtest2t_l2 pcovtest14t

rename pcovtest2e_l2 pcovtest14e

rename phqua2_l2 phqua14

rename pgrippe2_l2 pgrippe14

rename pcovtest2_l3 pcovtest15

rename pcovtest2m_l3 pcovtest15m

rename pcovtest2t_l3 pcovtest15t

rename pcovtest2e_l3 pcovtest15e

rename phqua2_l3 phqua15

rename pgrippe2_l3 pgrippe15

* Version-specific differences in item about home quaratine

label define phqua_v2 1 "Ja, verordnet" 2 "Ja, freiwillig" 3 "Nein"

forval i = 1/15 {

gen phqua_v2_`i' = phqua`i'

label val phqua_v2_`i' phqua_v2

replace phqua_v2_`i' = . if tranche == 1

}

label define phqua_v1 1 "Ja" 2 "Nein"

forval i = 1/15 {

replace phqua`i' = 1 if phqua`i' == 2 & tranche > 1

replace phqua`i' = 2 if phqua`i' == 3 & tranche > 1

label val phqua`i' phqua_v1

}

* Information on prevalence on household level

egen hh_test = anymatch(pcovtest1 pcovtest2 pcovtest3 pcovtest4 pcovtest5 pcovtest6 pcovtest7 pcovtest8 pcovtest9 pcovtest10 pcovtest11 pcovtest12 pcovtest13 pcovtest14 pcovtest15), values(1)

egen hh_test_count = anycount(pcovtest1 pcovtest2 pcovtest3 pcovtest4 pcovtest5 pcovtest6 pcovtest7 pcovtest8 pcovtest9 pcovtest10 pcovtest11 pcovtest12 pcovtest13 pcovtest14 pcovtest15), values(1)

egen hh_positive = anymatch(pcovtest1e pcovtest2e pcovtest3e pcovtest4e pcovtest5e pcovtest6e pcovtest7e pcovtest8e pcovtest9e pcovtest10e pcovtest11e pcovtest12e pcovtest13e pcovtest14e pcovtest15e), values(1)

egen hh_positive_count = anycount(pcovtest1e pcovtest2e pcovtest3e pcovtest4e pcovtest5e pcovtest6e pcovtest7e pcovtest8e pcovtest9e pcovtest10e pcovtest11e pcovtest12e pcovtest13e pcovtest14e pcovtest15e), values(1)

egen hh_hqua1 = anymatch(phqua1 phqua2 phqua3 phqua4 phqua5 phqua6 phqua7 phqua8 phqua9 phqua10 phqua11 phqua12 phqua13 phqua14 phqua15), values(1)

egen hh_hqua1_count = anycount(phqua1 phqua2 phqua3 phqua4 phqua5 phqua6 phqua7 phqua8 phqua9 phqua10 phqua11 phqua12 phqua13 phqua14 phqua15), values(1)

egen hh_grippe = anymatch(pgrippe1 pgrippe2 pgrippe3 pgrippe4 pgrippe5 pgrippe6 pgrippe7 pgrippe8 pgrippe9 pgrippe10 pgrippe11 pgrippe12 pgrippe13 pgrippe14 pgrippe15), values(1)

egen hh_grippe_count = anycount(pgrippe1 pgrippe2 pgrippe3 pgrippe4 pgrippe5 pgrippe6 pgrippe7 pgrippe8 pgrippe9 pgrippe10 pgrippe11 pgrippe12 pgrippe13 pgrippe14 pgrippe15), values(1)

foreach var in hh_test hh_test_count hh_positive hh_positive_count hh_hqua1 hh_hqua1_count hh_grippe hh_grippe_count {

replace `var' = . if syear != 2020

}

label variable hh_test "Coronavirus test "

label variable hh_test_count "Number of tested household members"

label variable hh_positive "Pos. tests "

label variable hh_positive_count "Number of household members with positive test"

label variable hh_hqua1 "Household members in quarantine"

label variable hh_hqua1_count "Nummer of household members in quarantine"

label variable hh_grippe "Flue symptoms "

label variable hh_grippe_count "Number of household members with flue symptoms"

label variable p_severeill "Propability mortal illness"

label variable d_severeill "Alreday having a mortal illness "

preserve

keep if syear == 2020

keep pid hh_test hh_positive p_severeill d_severeill

mvdecode _all , mv(-1=.a \ -2=.b \ -3 =.c \ -4 =.d \ -5=.e \ -6=.f \-7=.g \-8=.h)

tempfile temp1

save "`temp1'"

restore

* keep tranches with relevant info on vaccination

sum tranche if pcovimpf1!=.

keep if tranche>=r(min)

drop if tranche==.

keep pcovimpf* pid bula_cov

mvdecode _all , mv(-1=.a \ -2=.b \ -3 =.c \ -4 =.d \ -5=.e \ -6=.f \-7=.g \-8=.h)

save "$Data_prep\vaccination.dta", replace

* merge socio-demographic controls from previous years

use "$Data\soep_cov_${covversion}_statav13.dta", clear

keep if inrange(syear,2017,2019)

local controls "pla0009_v2 psample plh0204_v2 plh0216 plh0221 plh0226 plh0213 plh0219 plh0223 plh0215 plh0220 plh0225 plh0255 plh0214 plh0217 plh0224 plh0212 plh0218 plh0222 hghinc sex gebjahr plh0035 hlc0005_h hnetto hlk0044 plh0004 ple0008 pgpsbil bula bjk_87_02_* pgcasmin ple0012 ple0013 ple0014 ple0015 ple0016 ple0018 ple0020 ple0021 height weight fweight fheight hgtyp1hh"

foreach v of local controls{

local l`v' : variable label `v'

}

/* save the value labels for variables in local list*/

foreach var of local list{

levelsof `var', local(`var'_levels) /* create local list of all values of `var' */

foreach val of local `var'_levels { /* loop over all values in local list `var'_levels */

local `var'vl`val' : label `var' `val' /* create macro that contains label for each value */

}

}

keep pid syear `controls'

mvdecode _all , mv(-1=.a \ -2=.b \ -3 =.c \ -4 =.d \ -5=.e \ -6=.f \-7=.g \-8=.h)

reshape wide `controls', i(pid) j(syear)

/* apply the variable & value labels as variable labels */

/* variables are in form answeryear incyear */

foreach variable of local controls{

foreach value of local year_levels{

label variable `variable'`value' "`l`variable'': `yearvl`value''"

}

}

* factor analysis for BIG 5

foreach var of varlist plh02182019 plh02232019 plh02142019 plh02262019 {

replace `var' = (`var' -8) * -1

}

factor plh02162019 plh02212019 plh02262019 plh02132019 plh02192019 plh02232019 plh02152019 plh02202019 plh02252019 plh02552019 plh02142019 plh02172019 plh02242019 plh02122019 plh02182019 plh02222019 if !(psample2019 == 22 | psample2019 == 23 | inrange(psample2019,17,19)) & !mi(psample2019), factor(5)

rotate

predict extra consc open neuro agree if e(sample)

*********

rename plh0204_v22019 risk

*******

gen D_female=.

replace D_female=1 if pla0009_v22019==2 | sex2018==2

replace D_female=0 if pla0009_v22019==1 | sex2018==1

* Household net Y

gen vpi2018 = 103.8

gen vpi2019 = 105.3

gen nettoy=.

replace nettoy=hlc0005_h2019 *(100/vpi2019) if !mi(hlc0005_h2019)

replace nettoy=hlc0005_h2018 * (100/vpi2018) if nettoy==. & !mi(hlc0005_h2018)

replace nettoy=hghinc2018*(100/vpi2018) if nettoy==.

replace nettoy = nettoy / 1000

* number of kids

* number of kids < 16 (in 2019)

*cap drop N_kids16

*egen N_kids16= rownonmiss(bjk_87_02_12019 bjk_87_02_22019 bjk_87_02_32019 bjk_87_02_42019 bjk_87_02_52019 bjk_87_02_62019 bjk_87_02_72019 bjk_87_02_82019 bjk_87_02_92019)

*replace N_kids16 = 1 if N_kids16 > 0 &!mi(N_kids16)

*replace N_kids16 = 0 if N_kids16 == 0

gen byte N_kids16 = 1 if hlk00442019 == 1

replace N_kids16 = 0 if hlk00442019 == 2

* no single household

gen no_single=0

replace no_single=1 if hgtyp1hh2018 ==2 | hgtyp1hh2018 ==4 | hgtyp1hh2018 ==5 | hgtyp1hh2018 ==6 | hgtyp1hh2018 ==7 | hgtyp1hh2018 ==8

* Age

gen age=.

replace age=2020-gebjahr2018

gen D_age_29_low=0

replace D_age_29_low=1 if age<=29

gen D_age_30_49=0

replace D_age_30_49=1 if age>29 & age <= 49

gen D_age_50_69=0

replace D_age_50_69=1 if age>49 & age <= 69

gen D_age_70_plus=0

replace D_age_70_plus=1 if age>70 & age !=.

* Party preference (strong left / right)

/*

. tab plh00042019

2019 plh0004 | Freq. Percent Cum.

----------------------------------------+-----------------------------------

[0] 0 ganz links | 351 1.41 1.41

[1] 1 | 469 1.89 3.30

[2] 2 | 1,938 7.81 11.11

[3] 3 | 3,267 13.16 24.27

[4] 4 | 2,819 11.36 35.63

[5] 5 | 10,907 43.94 79.57

[6] 6 | 2,351 9.47 89.05

[7] 7 | 1,731 6.97 96.02

[8] 8 | 755 3.04 99.06

[9] 9 | 110 0.44 99.50

[10] 10 ganz rechts | 123 0.50 100.00

----------------------------------------+-----------------------------------

Total | 24,821 100.00

*/

gen byte D_left= 1 if inrange(plh00042019,0,4)

replace D_left = 0 if inrange(plh00042019,5,10)

gen byte D_middle=1 if plh00042019 == 5

replace D_middle = 0 if inrange(plh00042019,0,4) | inrange(plh00042019,6,10)

gen byte D_right= 0 if inrange(plh00042019,0,5)

replace D_right = 1 if inrange(plh00042019,6,10)

gen more_left=0

replace more_left=1 if D_left==1

replace more_left=-1 if D_right==1

* Education

cap drop degree

gen degree = 1 if inrange(pgcasmin2018,0,3)

replace degree = 2 if inrange(pgcasmin2018,4,7)

replace degree = 3 if inrange(pgcasmin2018,8,9)

replace degree = 1 if inrange(pgcasmin2017,0,3) & mi(degree)

replace degree = 2 if inrange(pgcasmin2017,4,7) & mi(degree)

replace degree = 3 if inrange(pgcasmin2017,8,9) & mi(degree)

label var degree "Educational degree"

label define degreel 1 "[1] CASMIN 0-3" 2 "[2] CASMIN 4-7" 3 "[3] CASMIN 8-9"

label val degree degreel

tab degree if !mi(degree), gen(d_degree)

gen education=0 if degree==1

replace education=0 if degree==2

replace education=1 if degree==3

* health status

* in general

gen health=.

replace health= ple00082019 if !mi(ple00082019)

replace health= ple00082018 if !mi(ple00082018) & mi(health)

**** Body weight and adipositas ****

gen bmi = weight2018 / ((height2018/100)^2) if !(fweight2018 == 1 | fheight2018 == 1)

label variable bmi "Body Mass Index"

gen byte adipositas = .

replace adipositas = 1 if bmi >= 40 & !mi(bmi)

replace adipositas = 0 if bmi <40 & !mi(bmi)

label var adipositas "Adipositas (2018er Werte in 2020)"

label define adipositasl 0 "[0] No Adipositas" 1 "[1] Adipositas"

label val adipositas adipositasl

**** Body weight and conditions ****

label define condl 0 "[0] No" 1 "[1] Yes"

* forward imputation of Diseases

foreach var of varlist ple00122019 ple00132019 ple00142019 ple00152019 ple00162019 ple00182019 ple00202019 ple00212019{

replace `var' = 0 if `var' == .b

label val `var' condl

}

rename ple00122019 d_diabetes

label var d_diabetes "Diabetes (2019)"

rename ple00132019 d_asthma

label var d_asthma "Asthma (2019)"

rename ple00142019 d_heart_dis

label var d_heart_dis "Heart disease (2019)"

rename ple00152019 d_cancer

label var d_cancer "Cancer (2019)"

rename ple00162019 d_stroke

label var d_stroke "Stroke (2019)"

rename ple00182019 d_blood_pressure

label var d_blood_pressure "High blood pressure (2019)"

rename ple00202019 d_dementia

label var d_dementia "Dementia (2019)"

rename ple00212019 d_joints

label var d_joints "Joint illness (2019)"

* number of parallel deseases

gen num_deseases_19=0

local list "d_diabetes d_asthma d_heart_dis d_cancer d_stroke d_blood_pressure d_dementia d_joints adipositas"

foreach var in `list' {

replace num_deseases_19=num_deseases_19+`var'

}

label var adipositas "Adipositas (2018)"

label var health "SRHS (2019/18)"

label var d_degree1 "CASMIN 0-3"

label var d_degree2 "CASMIN 4-7"

label var d_degree3 "CASMIN 8-9"

label var D_female "Female"

label var nettoy "Net income (2019/18)"

label var D_age_30_49 "Age 30-49"

label var D_age_50_69 "Age 50-69"

label var D_age_70_plus "Age >=70"

label var N_kids16 "Children below 16 (2019)"

label var D_left "Left spectrum"

label var D_middle "Centric spectrum"

label var D_right "Right spectrum"

label var extra "Extraversion (2019)"

label var consc "Conscientiousness (2019)"

label var open "Openness to experience (2019)"

label var neuro "Neuroticism (2019)"

label var agree "Agreeableness (2019)"

label var risk "WTR 2019"

gen health_n = health

gen risk_n = risk

save "$Data_prep\controls.dta", replace

use "$Data_prep\vaccination.dta", clear

merge 1:1 pid using "$Data_prep\controls.dta", keep(1 3) nogen

save "$Data_prep\vaccination.dta", replace

* defining dependent variables for regressions

use "$Data_prep\vaccination.dta", clear

* dummies for willingness to vaccination / obigatory vacination

gen Dum_voluntary=.

replace Dum_voluntary=1 if pcovimpf1==1

replace Dum_voluntary=0 if pcovimpf1==2

gen Dum_obligate=.

replace Dum_obligate=1 if pcovimpf2==1

replace Dum_obligate=0 if pcovimpf2==2

save "$Data_prep\vaccination.dta", replace

******* Preparation of weights ******

use "$Data\soep_cov_${covversion}_statav13.dta", clear

keep if tranche !=.

* define analysis population (people who got the vaccination questions

gen pop_vaccinate=0

sum tranche if pcovimpf1!=.

replace pop_vaccinate=1 if tranche >=r(min)

keep pid pop_vaccinate bula_cov pcovimpf1 pcovimpf2

save "$Data_prep\SOEP_cov_pop.dta", replace

merge 1:1 pid using "$Data_prep\controls.dta", keep(1 3) nogen

merge 1:1 pid using "`temp1'", keepusing(hh_test hh_positive p_severeill d_severeill) nogen keep(1 3)

*Region east west

gen byte D_east= 1 if inrange(bula_cov,11,16)

replace D_east = 0 if inrange(bula_cov,1,10)

label var D_east "Former GDR"

merge 1:1 pid using "$Data\2020-07-22_SOEP_CoV_Gewichte_v13.dta", keep(1 3) keepusing(phrf_cati) nogen

* statistically explain if respondent belongs to analysis population

local righthand "extra consc open neuro agree risk D_female D_age_30_49 D_age_50_69 D_age_70_plus d_degree2 d_degree3 nettoy N_kids16 D_east health more_left num_deseases_19 hh_test hh_positive p_severeill"

logit pop_vaccinate `righthand'

/*

mat b = r(table)

local dim = colsof(b) - 1

di `dim'

local pred ""

forval i = 1(1)`dim'{

if (abs(b[1,`i']) > 0.01 & b[4,`i'] < 0.05){

di "hello `i'"

local intm : word `i' of `righthand'"

local pred "`pred' `intm'"

}

else{

}

}

su `pred'

* If a variable is significant (p < 0.05) AND relevant (beta>0.01), weights need not be adjusted

*/

* Otherwise:

* (1) re-run logit with !significant AND relevant! variables

local righthandrel "extra hh_test"

logit pop_vaccinate `righthandrel'

* (2) predict

predict pred

* (3) compute inverse probability

gen w_vacc=1/pred

* (4) Multiply weight with inverse probability

replace w_vacc=w_vacc * phrf_cati

replace w_vacc=phrf_cati if mi(w_vacc)

* (5) keep relevant variables

keep pid w_vacc

*/

*ren phrf_cati weight

ren w_vacc weight

save "$Data_prep\vaccination_weights.dta", replace

* Merge new weight with "$Data_prep\vaccination.dta"

use "$Data_prep\vaccination.dta", replace

*east west

gen byte D_east= 1 if inrange(bula_cov,11,16)

replace D_east = 0 if inrange(bula_cov,1,10)

label var D_east "Former GDR"

merge 1:1 pid using "$Data_prep\vaccination_weights.dta", keep(1 3) keepusing(weight) nogen

replace weight = round(weight)

merge 1:1 pid using "`temp1'", keepusing(hh_test hh_positive p_severeill d_severeill) nogen keep(1 3)

* I rescale variables without natural scale to hav mean zero and standard deviation

foreach var in extra consc open neuro agree risk health more_left{

su `var'

replace `var' = (`var'-r(mean))/r(sd)

}

save "$Data_prep\vaccination.dta", replace

*** Merge date of interview

use pid pcovimpf1 syear tranche pdatt pdatm using "$Data\soep_cov_${covversion}_statav13.dta", clear

sum tranche if pcovimpf1==1

keep if tranche>=r(min)

keep if tranche!=.

keep pdatt pdatm pid

merge 1:1 pid using "$Data_prep\vaccination.dta"

drop _merge

save "$Data_prep\vaccination.dta", replace

*************************************

************ ANALYSIS ***************

*************************************

use "$Data_prep\vaccination.dta", clear

* Basic info about sample

sum pdatm

scalar sc_month_begin=r(min)

scalar sc_month_end=r(max)

scalar sc_N=r(N)

disp "Grom month " sc_month_begin " to " sc_month_end " , " sc_N " respondents participated in the vaccination module of SOEP-CoV "

* cross tab voluntary vacc / vacc obligation

tabulate pcovimpf1 pcovimpf2 [w=weight] if (pcovimpf1!=-1 & pcovimpf2!=-1), cell nofreq

*** arguments across 4 groups (constructed by Dum_voluntary / Dum_obligatory)

use "$Data_prep\vaccination.dta", clear

gen group=0

replace group=1 if Dum_voluntary==0 & Dum_obligate==0

replace group=2 if Dum_voluntary==1 & Dum_obligate==0

replace group=3 if Dum_voluntary==0 & Dum_obligate==1

replace group=4 if Dum_voluntary==1 & Dum_obligate==1

keep if group>0

svyset pid [pweight=weight]

gen argument_1=0

replace argument_1= pcovimpf3 if pcovimpf3==1

replace argument_1= pcovimpf7 if pcovimpf7==1

gen argument_2=0

replace argument_2= pcovimpf4 if pcovimpf4==1

replace argument_2= pcovimpf8 if pcovimpf8==1

gen argument_3=0

replace argument_3= pcovimpf5 if pcovimpf5==1

replace argument_3= pcovimpf9 if pcovimpf9==1

gen argument_4=0

replace argument_4= pcovimpf6 if pcovimpf6==1

replace argument_4= pcovimpf10 if pcovimpf10==1

global argument "argument_1 argument_2 argument_3 argument_4"

foreach var of varlist $argument {

forvalues g=1(1)4 {

replace `var' = `var' * 100

sum `var' [w=weight] if group==`g'

scalar mu_`var'_`g'=r(mean)

replace `var' = `var' /100

}

replace `var' = `var' * 100

sum `var' [w=weight]

scalar mu_`var'_all=r(mean)

replace `var' = `var' /100

}

svyset pid [pweight=weight]

foreach var of varlist $argument {

svy: mean `var', over(group)

test [`var']1 = [`var']2

local F_`var'_1_2: di %5.3f r(p)

local p = `r(p)'

count_stars, p(`p')

local F_`var'_1_2: di "`F_`var'_1_2'`r(stars)'"

test [`var']1 = [`var']3

local F_`var'_1_3: di %5.3f r(p)

local p = `r(p)'

count_stars, p(`p')

local F_`var'_1_3: di "`F_`var'_1_3'`r(stars)'"

test [`var']1 = [`var']4

local F_`var'_1_4: di %5.3f r(p)

local p = `r(p)'

count_stars, p(`p')

local F_`var'_1_4: di "`F_`var'_1_4'`r(stars)'"

test [`var']2 = [`var']3

local F_`var'_2_3: di %5.3f r(p)

local p = `r(p)'

count_stars, p(`p')

local F_`var'_2_3: di "`F_`var'_2_3'`r(stars)'"

test [`var']2 = [`var']4

local F_`var'_2_4: di %5.3f r(p)

local p = `r(p)'

count_stars, p(`p')

local F_`var'_2_4: di "`F_`var'_2_4'`r(stars)'"

test [`var']3 = [`var']4

local F_`var'_3_4: di %5.3f r(p)

local p = `r(p)'

count_stars, p(`p')

local F_`var'_3_4: di "`F_`var'_3_4'`r(stars)'"

}

clear

set obs 4

input mu_all mu_group_1 mu_group_2 mu_group_3 mu_group_4 str4 F_stat_1_2 str4 F_stat_1_3 str4 F_stat_1_4 str4 F_stat_2_3 str4 F_stat_2_4 str4 F_stat_3_4

end

generate str Variable = "argument_1" in 1

replace Variable = "argument_2" in 2

replace Variable = "argument_3" in 3

replace Variable = "argument_4" in 4

order Variable

foreach var in $argument {

forvalues g=1(1)4 {

replace mu_all = round(mu_`var'_all, 0.1) if Variable=="`var'"

}

}

foreach var in $argument {

forvalues g=1(1)4 {

replace mu_group_`g' = mu_`var'_`g' if Variable=="`var'"

}

}

foreach var in $argument {

forvalues g=1(1)1 {

forvalues h=2(1)4 {

replace F_stat_`g'_`h' = "`F_`var'_`g'_`h''" if Variable=="`var'"

}

}

forvalues g=2(1)2 {

forvalues h=3(1)4 {

replace F_stat_`g'_`h' = "`F_`var'_`g'_`h''" if Variable=="`var'"

}

}

forvalues g=3(1)3 {

forvalues h=4(1)4 {

replace F_stat_`g'_`h' = "`F_`var'_`g'_`h''" if Variable=="`var'"

}

}

}

local vars "mu_group_1 mu_group_2 mu_group_3 mu_group_4"

foreach var of varlist `vars' {

replace `var'=round(`var', 0.1)

}

save "$tables\Table_4groups_arguments", replace

export excel using "$tables\Table_4groups_arguments", firstrow(variables) replace

***** Characteristics of four groups (constructed by Dum_voluntary/Dum_obligatory)

use "$Data_prep\vaccination.dta", clear

global vars "D_female age education nettoy N_kids16 D_east extra consc open neuro agree risk health num_deseases_19 hh_test hh_positive p_severeill more_left"

foreach var of varlist D_female education N_kids16 D_east hh_test hh_positive{

replace `var' = `var' * 100

}

gen group=0

replace group=1 if Dum_voluntary==0 & Dum_obligate==0

replace group=2 if Dum_voluntary==1 & Dum_obligate==0

replace group=3 if Dum_voluntary==0 & Dum_obligate==1

replace group=4 if Dum_voluntary==1 & Dum_obligate==1

keep if group>0

svyset pid [pweight=weight]

* using weights for ttest

svyset pid [pweight=weight]

foreach var of varlist $vars {

svy: mean `var', over(group)

test [`var']1 = [`var']2

local F_`var'_1_2: di %5.3f r(p)

local p = `r(p)'

count_stars, p(`p')

local F_`var'_1_2: di "`F_`var'_1_2'`r(stars)'"

test [`var']1 = [`var']3

local F_`var'_1_3: di %5.3f r(p)

local p = `r(p)'

count_stars, p(`p')

local F_`var'_1_3: di "`F_`var'_1_3'`r(stars)'"

test [`var']1 = [`var']4

local F_`var'_1_4: di %5.3f r(p)

local p = `r(p)'

count_stars, p(`p')

local F_`var'_1_4: di "`F_`var'_1_4'`r(stars)'"

test [`var']2 = [`var']3

local F_`var'_2_3: di %5.3f r(p)

local p = `r(p)'

count_stars, p(`p')

local F_`var'_2_3: di "`F_`var'_2_3'`r(stars)'"

test [`var']2 = [`var']4

local F_`var'_2_4: di %5.3f r(p)

local p = `r(p)'

count_stars, p(`p')

local F_`var'_2_4: di "`F_`var'_2_4'`r(stars)'"

test [`var']3 = [`var']4

local F_`var'_3_4: di %5.3f r(p)

local p = `r(p)'

count_stars, p(`p')

local F_`var'_3_4: di "`F_`var'_3_4'`r(stars)'"

}

foreach var of varlist $vars {

forvalues g=1(1)4 {

sum `var' [w=weight] if group==`g'

scalar mu_`var'_`g'=r(mean)

}

sum `var' [w=weight]

scalar mu_`var'_all = r(mean)

}

clear

local obs : list sizeof global(vars)

set obs `obs'

input mu_all mu_group_1 mu_group_2 mu_group_3 mu_group_4 str6 F_stat_1_2 str6 F_stat_1_3 str6 F_stat_1_4 str6 F_stat_2_3 str6 F_stat_2_4 str6 F_stat_3_4

end

gen str Variable = "."

local num = 0

foreach var in $vars{

local num = `num' + 1

replace Variable = "`var'" in `num'

}

foreach var in $vars {

forvalues g=1(1)4 {

replace mu_group_`g' = mu_`var'_`g' if Variable=="`var'"

}

}

foreach var in $vars {

replace mu_all = mu_`var'_all if Variable=="`var'"

}

foreach var in $vars {

forvalues g=1(1)1 {

forvalues h=2(1)4 {

replace F_stat_`g'_`h' = "`F_`var'_`g'_`h''" if Variable=="`var'"

}

}

forvalues g=2(1)2 {

forvalues h=3(1)4 {

replace F_stat_`g'_`h' = "`F_`var'_`g'_`h''" if Variable=="`var'"

}

}

forvalues g=3(1)3 {

forvalues h=4(1)4 {

replace F_stat_`g'_`h' = "`F_`var'_`g'_`h''" if Variable=="`var'"

}

}

}

local vars "mu_all mu_group_1 mu_group_2 mu_group_3 mu_group_4"

foreach var of varlist `vars' {

replace `var'=round(`var', 0.01)

}

save "$tables\Groups_characteristics", replace

export excel using "$tables\Groups_characteristics", firstrow(variables) replace

*** Explaining voluntary vaccination ****

use "$Data_prep\vaccination.dta", clear

global righthand "i.D_female age i.education nettoy i.N_kids16 i.D_east extra consc open neuro agree risk health num_deseases_19 i.hh_test i.hh_positive p_severeill more_left"

logit Dum_voluntary $righthand, vce(robust)

margins, dydx(*) post

outreg2 using "${tables}wtv_mfx2", word replace long ctitle(WTV voluntarly) label bdec(3)

preserve

regsave, ci pval tstat

foreach var in 0b.D_female 0b.education 0b.N_kids16 0b.D_east 0b.hh_test 0b.hh_positive{

drop if var == "`var'"

}

keep coef stderr pval ci_lower ci_upper tstat

format _all %5.3f

order coef stderr ci_lower ci_upper tstat pval

mkmat _all, mat(vol)

restore

logit Dum_obligate $righthand, vce(robust)

margins, dydx(*) post

outreg2 using "${tables}wtv_mfx2", word append long ctitle(WTV mandatory) label bdec(3)

preserve

regsave, ci pval tstat

foreach var in 0b.D_female 0b.education 0b.N_kids16 0b.D_east 0b.hh_test 0b.hh_positive{

drop if var == "`var'"

}

keep coef stderr pval ci_lower ci_upper tstat

format _all %5.3f

order coef stderr ci_lower ci_upper tstat pval

mkmat _all, mat(obl)

restore

putexcel set ${tables}plos_one_results.xlsx, modify sheet(Voluntary)

putexcel B3 = matrix(vol), nformat("0.000")

putexcel close

putexcel set ${tables}plos_one_results.xlsx, modify sheet(Obligatory)

putexcel B3 = matrix(obl), nformat("0.000")

putexcel close

*****************************************************

************* Additions for revision ****************

*****************************************************

*****************************************************

**** Dropping non-significant characteristics *******

*****************************************************

use "$Data_prep\vaccination.dta", clear

global righthand "i.D_female age i.education nettoy i.N_kids16 i.D_east extra consc open neuro agree risk health num_deseases_19 i.hh_test i.hh_positive p_severeill more_left"

logit Dum_voluntary $righthand, vce(robust)

global righthandnonsig "i.D_female age i.education nettoy p_severeill"

logit Dum_voluntary $righthandnonsig if e(sample) == 1, vce(robust)

margins, dydx(*) post

use "$Data_prep\vaccination.dta", clear

global righthand "i.D_female age i.education nettoy i.N_kids16 i.D_east extra consc open neuro agree risk health num_deseases_19 i.hh_test i.hh_positive p_severeill more_left"

logit Dum_obligate $righthand, vce(robust)

margins, dydx(*) post

global righthandnonsig "i.D_female age i.D_east neuro p_severeill"

logit Dum_obligate $righthandnonsig if e(sample) == 1, vce(robust)

margins, dydx(*) post

*****************************************************

***************** ROC analysis **********************

*****************************************************

use "$Data_prep\vaccination.dta", clear

global righthand "i.D_female age i.education nettoy i.N_kids16 i.D_east extra consc open neuro agree risk health num_deseases_19 i.hh_test i.hh_positive p_severeill more_left"

logit Dum_voluntary $righthand, vce(robust)

lroc, scheme(s1mono)

graph export ${figures}roc_curve_logit_voluntary_vaccination.png, as(png) replace

logit Dum_obligate $righthand, vce(robust)

lroc, scheme(s1mono)

graph export ${figures}lroc_obligate.png, as(png) replace

graph export ${figures}roc_curve_logit_obligatory_vaccination.png, as(png) replace

*****************************************************

********************* Linktest **********************

*****************************************************

use "$Data_prep\vaccination.dta", clear

global righthand "i.D_female age i.education nettoy i.N_kids16 i.D_east extra consc open neuro agree risk health num_deseases_19 i.hh_test i.hh_positive p_severeill more_left"

logit Dum_voluntary $righthand, vce(robust)

cap drop yh1

predict yh1, xb

gen yh12 = yh1^2

logit Dum_voluntary yh1 yh12

mat res1 = r(table)

logit Dum_obligate $righthand, vce(robust)

cap drop yh2

predict yh2, xb

gen yh22 = yh2^2

logit Dum_obligate yh2 yh22

mat res2 = r(table)

putexcel set ${tables}plos_one_link.xlsx, modify sheet(Voluntary)

putexcel A2 = "Explanatory variable"

putexcel A3 = "xb"

putexcel A4 = "(xb)^2"

putexcel B2 = "Effect"

putexcel C2 = "S.E."

putexcel D2 = "LB 95% CI"

putexcel E2 = "UB 95% CI"

putexcel F2 = "z-statistic"

putexcel G2 = "p-value"

putexcel B3 = res1[1,1], nformat(0.000)

putexcel C3 = res1[2,1], nformat(0.000)

putexcel D3 = res1[5,1], nformat(0.000)

putexcel E3 = res1[6,1], nformat(0.000)

putexcel F3 = res1[3,1], nformat(0.000)

putexcel G3 = res1[4,1], nformat(0.000)

putexcel B4 = res1[1,2], nformat(0.000)

putexcel C4 = res1[2,2], nformat(0.000)

putexcel D4 = res1[5,2], nformat(0.000)

putexcel E4 = res1[6,2], nformat(0.000)

putexcel F4 = res1[3,2], nformat(0.000)

putexcel G4 = res1[4,2], nformat(0.000)

putexcel close

putexcel set ${tables}plos_one_link.xlsx, modify sheet(Obligatory)

putexcel A2 = "Explanatory variable"

putexcel A3 = "xb"

putexcel A4 = "(xb)^2"

putexcel B2 = "Effect"

putexcel C2 = "S.E."

putexcel D2 = "LB 95% CI"

putexcel E2 = "UB 95% CI"

putexcel F2 = "z-statistic"

putexcel G2 = "p-value"

putexcel B3 = res2[1,1], nformat(0.000)

putexcel C3 = res2[2,1], nformat(0.000)

putexcel D3 = res2[5,1], nformat(0.000)

putexcel E3 = res2[6,1], nformat(0.000)

putexcel F3 = res2[3,1], nformat(0.000)

putexcel G3 = res2[4,1], nformat(0.000)

putexcel B4 = res2[1,2], nformat(0.000)

putexcel C4 = res2[2,2], nformat(0.000)

putexcel D4 = res2[5,2], nformat(0.000)

putexcel E4 = res2[6,2], nformat(0.000)

putexcel F4 = res2[3,2], nformat(0.000)

putexcel G4 = res2[4,2], nformat(0.000)

putexcel close

*****************************************************

***** Variance inflation factor *********************

*****************************************************

use "$Data_prep\vaccination.dta", clear

global righthand "i.D_female age i.education nettoy i.N_kids16 i.D_east extra consc open neuro agree risk health num_deseases_19 i.hh_test i.hh_positive p_severeill more_left"

putexcel set ${tables}plos_one_vif.xlsx, modify sheet(Voluntary)

putexcel B5 = "Female"

putexcel B6 = "Age"

putexcel B7 = "Tertiary education"

putexcel B8 = "Net monthly income per household, 1k EUR"

putexcel B9 = "Children younger than 17"

putexcel B10 = "Eastern federal states"

putexcel B11 = "Extraversion"

putexcel B12 = "Conscientiousness"

putexcel B13 = "Openness to experience"

putexcel B14 = "Neuroticism"

putexcel B15 = "Agreeableness"

putexcel B16 = "Willingness to take risks"

putexcel B17 = "Health: Self-assessment"

putexcel B18 = "Number of risk diseases"

putexcel B19 = "Test for COVID-19 in household"

putexcel B20 = "Positive test for COVID-19 in household"

putexcel B21 = "Prob. of life-threatening disease (in %)"

putexcel B22 = "Political preferences"

logit Dum_voluntary $righthand, vce(robust)

vif, uncentered

forval i = 1(1)18{

local h = 4 + `i'

local s: di %5.3f r(vif_`i')

di `s'

putexcel C`h' = `s'

}

*/

putexcel close

putexcel set ${tables}plos_one_vif.xlsx, modify sheet(Obligatory)

putexcel B5 = "Female"

putexcel B6 = "Age"

putexcel B7 = "Tertiary education"

putexcel B8 = "Net monthly income per household, 1k EUR"

putexcel B9 = "Children younger than 17"

putexcel B10 = "Eastern federal states"

putexcel B11 = "Extraversion"

putexcel B12 = "Conscientiousness"

putexcel B13 = "Openness to experience"

putexcel B14 = "Neuroticism"

putexcel B15 = "Agreeableness"

putexcel B16 = "Willingness to take risks"

putexcel B17 = "Health: Self-assessment"

putexcel B18 = "Number of risk diseases"

putexcel B19 = "Test for COVID-19 in household"

*putexcel B20 = "Positive test for COVID-19 in household"

putexcel B20 = "Prob. of life-threatening disease (in %)"

putexcel B21 = "Political preferences"

logit Dum_obligate $righthand, vce(robust)

vif, uncentered

forval i = 1(1)17{

local h = 4 + `i'

local s: di %5.3f r(vif_`i')

di `s'

putexcel C`h' = `s'

}

*/

putexcel close

*** logit model without age ****

use "$Data_prep\vaccination.dta", clear

global righthand2 "i.D_female i.education nettoy i.N_kids16 i.D_east extra consc open neuro agree risk health num_deseases_19 i.hh_test i.hh_positive p_severeill more_left"

global righthand "i.D_female age i.education nettoy i.N_kids16 i.D_east extra consc open neuro agree risk health num_deseases_19 i.hh_test i.hh_positive p_severeill more_left"

logit Dum_voluntary $righthand, vce(robust)

logit Dum_voluntary $righthand2 if e(sample) == 1, vce(robust)

logit Dum_voluntary $righthand2 if e(sample) == 1, vce(robust)

margins, dydx(*) post

outreg2 using "${tables}wtv_mfx2_vif_age", word replace long ctitle(WTV voluntarly) label bdec(3)

preserve

regsave, ci pval tstat

foreach var in 0b.D_female 0b.education 0b.N_kids16 0b.D_east 0b.hh_test 0b.hh_positive{

drop if var == "`var'"

}

keep coef stderr pval ci_lower ci_upper tstat

format _all %5.3f

order coef stderr ci_lower ci_upper tstat pval

mkmat _all, mat(vol)

restore

logit Dum_obligate $righthand, vce(robust)

logit Dum_obligate $righthand2 if e(sample) == 1, vce(robust)

margins, dydx(*) post

outreg2 using "${tables}wtv_mfx2_vif_age", word append long ctitle(WTV mandatory) label bdec(3)

preserve

regsave, ci pval tstat

foreach var in 0b.D_female 0b.education 0b.N_kids16 0b.D_east 0b.hh_test 0b.hh_positive{

drop if var == "`var'"

}

keep coef stderr pval ci_lower ci_upper tstat

format _all %5.3f

order coef stderr ci_lower ci_upper tstat pval

mkmat _all, mat(obl)

restore

putexcel set ${tables}plos_one_results_vif_age.xlsx, modify sheet(Voluntary)

putexcel B3 = matrix(vol), nformat("0.000")

putexcel close

putexcel set ${tables}plos_one_results_vif_age.xlsx, modify sheet(Obligatory)

putexcel B3 = matrix(obl), nformat("0.000")

putexcel close

*****************************************************

*********Imputation of missings *********************

*****************************************************

use "$Data_prep\vaccination.dta", clear

global righthand "i.D_female age i.education nettoy i.N_kids16 i.D_east extra consc open neuro agree risk health num_deseases_19 i.hh_test i.hh_positive p_severeill more_left"

gen Dum_voluntary_r=Dum_voluntary

replace Dum_voluntary_r=1 if Dum_voluntary==.

logit Dum_voluntary_r $righthand, vce(robust)

margins, dydx(*) post

outreg2 using "${tables}impute_voluntary", word replace long ctitle(Imputed voluntarily) label bdec(3)

preserve

regsave, ci pval tstat

foreach var in 0b.D_female 0b.education 0b.N_kids16 0b.D_east 0b.hh_test 0b.hh_positive{

drop if var == "`var'"

}

keep coef stderr pval ci_lower ci_upper tstat

format _all %5.3f

order coef stderr ci_lower ci_upper tstat pval

mkmat _all, mat(vol_imp)

restore

* b) compulsory vaccination question: Assigning refusing respondents to "yes" group

gen Dum_obligate_r=Dum_obligate

replace Dum_obligate_r=1 if Dum_obligate==.

logit Dum_obligate_r $righthand, vce(robust)

margins, dydx(*) post

outreg2 using "${tables}impute_obligate", word replace long ctitle(Imputed obligatory) label bdec(3)

preserve

regsave, ci pval tstat

foreach var in 0b.D_female 0b.education 0b.N_kids16 0b.D_east 0b.hh_test 0b.hh_positive{

drop if var == "`var'"

}

keep coef stderr pval ci_lower ci_upper tstat

format _all %5.3f

order coef stderr ci_lower ci_upper tstat pval

mkmat _all, mat(obl_imp)

restore

putexcel set ${tables}plos_one_results_imputed.xlsx, modify sheet(Voluntary)

putexcel B3 = matrix(vol_imp), nformat("0.000")

putexcel close

putexcel set ${tables}plos_one_results_imputed.xlsx, modify sheet(Obligatory)

putexcel B3 = matrix(obl_imp), nformat("0.000")

putexcel close

*****************************************************

***** Comparing variances across groups *************

*****************************************************

use "$Data_prep\vaccination.dta", clear

global vars "D_female age education nettoy N_kids16 D_east extra consc open neuro agree risk health num_deseases_19 hh_test p_severeill more_left"

foreach var of varlist D_female education N_kids16 D_east hh_test hh_positive{

replace `var' = `var' * 100

}

gen group=0

replace group=1 if Dum_voluntary==0 & Dum_obligate==0

replace group=2 if Dum_voluntary==1 & Dum_obligate==0

replace group=3 if Dum_voluntary==0 & Dum_obligate==1

replace group=4 if Dum_voluntary==1 & Dum_obligate==1

keep if group>0

svyset pid [pweight=weight]

* equality test

foreach var in $vars{

gen `var'_sq = `var'* `var'

svy : mean `var'*, over(group)

testnl ( _b[`var'_sq:1] - _b[`var':1]*_b[`var':1]) ///

= ( _b[`var'_sq:2] - _b[`var':2]*_b[`var':2])

local chi_`var'_1_2: di %5.3f r(p)

local p = `r(p)'

count_stars, p(`p')

local chi_`var'_1_2: di "`chi_`var'_1_2'"

testnl ( _b[`var'_sq:1] - _b[`var':1]*_b[`var':1]) ///

= ( _b[`var'_sq:3] - _b[`var':3]*_b[`var':3])

local chi_`var'_1_3: di %5.3f r(p)

local p = `r(p)'

count_stars, p(`p')

local chi_`var'_1_3: di "`chi_`var'_1_3'"

testnl ( _b[`var'_sq:1] - _b[`var':1]*_b[`var':1]) ///

= ( _b[`var'_sq:4] - _b[`var':4]*_b[`var':4])

local chi_`var'_1_4: di %5.3f r(p)

local p = `r(p)'

count_stars, p(`p')

local chi_`var'_1_4: di "`chi_`var'_1_4'"

testnl ( _b[`var'_sq:2] - _b[`var':2]*_b[`var':2]) ///

= ( _b[`var'_sq:3] - _b[`var':3]*_b[`var':3])

local chi_`var'_2_3: di %5.3f r(p)

local p = `r(p)'

count_stars, p(`p')

local chi_`var'_2_3: di "`chi_`var'_2_3'"

testnl ( _b[`var'_sq:2] - _b[`var':2]*_b[`var':2]) ///

= ( _b[`var'_sq:4] - _b[`var':4]*_b[`var':4])

local chi_`var'_2_4: di %5.3f r(p)

local p = `r(p)'

count_stars, p(`p')

local chi_`var'_2_4: di "`chi_`var'_2_4'"

testnl ( _b[`var'_sq:3] - _b[`var':3]*_b[`var':3]) ///

= ( _b[`var'_sq:4] - _b[`var':4]*_b[`var':4])

local chi_`var'_3_4: di %5.3f r(p)

local p = `r(p)'

count_stars, p(`p')

local chi_`var'_3_4: di "`chi_`var'_3_4'"

cap drop `var'_sq

}

clear

local obs : list sizeof global(vars)

set obs `obs'

input str6 chi_stat_1_2 str6 chi_stat_1_3 str6 chi_stat_1_4 str6 chi_stat_2_3 str6 chi_stat_2_4 str6 chi_stat_3_4

end

gen str Variable = "."

local num = 0

foreach var in $vars{

local num = `num' + 1

replace Variable = "`var'" in `num'

}

foreach var in $vars {

forvalues g=1(1)1 {

forvalues h=2(1)4 {

replace chi_stat_`g'_`h' = "`chi_`var'_`g'_`h''" if Variable=="`var'"

}

}

forvalues g=2(1)2 {

forvalues h=3(1)4 {

replace chi_stat_`g'_`h' = "`chi_`var'_`g'_`h''" if Variable=="`var'"

}

}

forvalues g=3(1)3 {

forvalues h=4(1)4 {

replace chi_stat_`g'_`h' = "`chi_`var'_`g'_`h''" if Variable=="`var'"

}

}

}

save "$tables\Groups_characteristics_var", replace

export excel using "$tables\Groups_characteristics_var", firstrow(variables) replace

use "$Data_prep\vaccination.dta", clear

global vars "D_female age education nettoy N_kids16 D_east extra consc open neuro agree risk health num_deseases_19 hh_test p_severeill more_left"

foreach var of varlist D_female education N_kids16 D_east hh_test hh_positive{

replace `var' = `var' * 100

}

gen group=0

replace group=1 if Dum_voluntary==0 & Dum_obligate==0

replace group=2 if Dum_voluntary==1 & Dum_obligate==0

replace group=3 if Dum_voluntary==0 & Dum_obligate==1

replace group=4 if Dum_voluntary==1 & Dum_obligate==1

keep if group>0

foreach var in $vars{

reg `var' i.group if group == 1 | group == 2 [pweight=weight], robust

mat b = r(table)

local p: di %5.3f b[4,2]

di `p'

count_stars, p(`p')

local t_`var'_1_2: di "`p'"

reg `var' i.group if group == 1 | group == 3 [pweight=weight], robust

mat b = r(table)

local p: di %5.3f b[4,2]

di `p'

count_stars, p(`p')

local t_`var'_1_3: di "`p'"

reg `var' i.group if group == 1 | group == 4 [pweight=weight], robust

mat b = r(table)

local p: di %5.3f b[4,2]

di `p'

count_stars, p(`p')

local t_`var'_1_4: di "`p'"

reg `var' i.group if group == 2 | group == 3 [pweight=weight], robust

mat b = r(table)

local p: di %5.3f b[4,2]

di `p'

count_stars, p(`p')

local t_`var'_2_3: di "`p'"

reg `var' i.group if group == 2 | group == 4 [pweight=weight], robust

mat b = r(table)

local p: di %5.3f b[4,2]

di `p'

count_stars, p(`p')

local t_`var'_2_4: di "`p'"

reg `var' i.group if group == 3 | group == 4 [pweight=weight], robust

mat b = r(table)

local p: di %5.3f b[4,2]

di `p'

count_stars, p(`p')

local t_`var'_3_4: di "`p'"

}

clear

local obs : list sizeof global(vars)

set obs `obs'

input str6 t_stat_1_2 str6 t_stat_1_3 str6 t_stat_1_4 str6 t_stat_2_3 str6 t_stat_2_4 str6 t_stat_3_4

end

gen str Variable = "."

local num = 0

foreach var in $vars{

local num = `num' + 1

replace Variable = "`var'" in `num'

}

foreach var in $vars {

forvalues g=1(1)1 {

forvalues h=2(1)4 {

replace t_stat_`g'_`h' = "`t_`var'_`g'_`h''" if Variable=="`var'"

}

}

forvalues g=2(1)2 {

forvalues h=3(1)4 {

replace t_stat_`g'_`h' = "`t_`var'_`g'_`h''" if Variable=="`var'"

}

}

forvalues g=3(1)3 {

forvalues h=4(1)4 {

replace t_stat_`g'_`h' = "`t_`var'_`g'_`h''" if Variable=="`var'"

}

}

}

save "$tables\Groups_characteristics_test_uneq", replace

export excel using "$tables\Groups_characteristics_test_uneq", firstrow(variables) replace

*****************************************************

***** Multivariate probit ***************************

*****************************************************

use "$Data_prep\vaccination.dta", clear

global righthand "D_female age education nettoy N_kids16 D_east extra consc open neuro agree risk health num_deseases_19 hh_test hh_positive p_severeill more_left"

probit Dum_voluntary Dum_obligate $righthand

keep if e(sample) == 1

mvprobit (Dum_obligate = $righthand) (Dum_voluntary = $righthand), vce(robust) seed(123456789)

preserve

regsave, ci pval tstat

keep in 1/18

keep coef stderr pval ci_lower ci_upper tstat

format _all %5.3f

order coef stderr ci_lower ci_upper tstat pval

mkmat _all, mat(obl_mvprob)

restore

preserve

regsave, ci pval tstat

keep in 19/36

keep coef stderr pval ci_lower ci_upper tstat

format _all %5.3f

order coef stderr ci_lower ci_upper tstat pval

mkmat _all, mat(vol_mvprob)

restore

global righthand "i.D_female age i.education nettoy i.N_kids16 i.D_east extra consc open neuro agree risk health num_deseases_19 i.hh_test p_severeill more_left"

probit Dum_voluntary $righthand, vce(robust)

preserve

regsave, ci pval tstat

foreach var in Dum_voluntary:0b.D_female Dum_voluntary:0b.education Dum_voluntary:0b.N_kids16 Dum_voluntary:0b.D_east Dum_voluntary:0b.hh_test Dum_voluntary:0b.hh_positive{

drop if var == "`var'"

}

keep coef stderr pval ci_lower ci_upper tstat

format _all %5.3f

order coef stderr ci_lower ci_upper tstat pval

mkmat _all, mat(vol_prob)

restore

margins, dydx(*) post

preserve

regsave, ci pval tstat

foreach var in 0b.D_female 0b.education 0b.N_kids16 0b.D_east 0b.hh_test 0b.hh_positive{

drop if var == "`var'"

}

keep coef stderr pval ci_lower ci_upper tstat

format _all %5.3f

order coef stderr ci_lower ci_upper tstat pval

mkmat _all, mat(vol_avgprob)

restore

probit Dum_obligate $righthand, vce(robust)

preserve

regsave, ci pval tstat

foreach var in Dum_obligate:0b.D_female Dum_obligate:0b.education Dum_obligate:0b.N_kids16 Dum_obligate:0b.D_east Dum_obligate:0b.hh_test Dum_obligate:0b.hh_positive{

drop if var == "`var'"

}

keep coef stderr pval ci_lower ci_upper tstat

format _all %5.3f

order coef stderr ci_lower ci_upper tstat pval

mkmat _all, mat(ob_prob)

restore

margins, dydx(*) post

preserve

regsave, ci pval tstat

foreach var in 0b.D_female 0b.education 0b.N_kids16 0b.D_east 0b.hh_test 0b.hh_positive{

drop if var == "`var'"

}

keep coef stderr pval ci_lower ci_upper tstat

format _all %5.3f

order coef stderr ci_lower ci_upper tstat pval

mkmat _all, mat(ob_avgprob)

restore

putexcel set ${tables}plos_one_mvprob.xlsx, sheet(MVObligatory) replace

putexcel B5 = "Female"

putexcel B6 = "Age"

putexcel B7 = "Tertiary education"

putexcel B8 = "Net monthly income per household, 1k EUR"

putexcel B9 = "Children younger than 17"

putexcel B10 = "Eastern federal states"

putexcel B11 = "Extraversion"

putexcel B12 = "Conscientiousness"

putexcel B13 = "Openness to experience"

putexcel B14 = "Neuroticism"

putexcel B15 = "Agreeableness"

putexcel B16 = "Willingness to take risks"

putexcel B17 = "Health: Self-assessment"

putexcel B18 = "Number of risk diseases"

putexcel B19 = "Test for COVID-19 in household"

putexcel B20 = "Prob. of life-threatening disease (in %)"

putexcel B21 = "Political preferences"

putexcel B22 = "Constant"

putexcel C4 = "Effect"

putexcel D4 = "S.E."

putexcel E4 = "LB 95% CI"

putexcel F4 = "UB 95% CI"

putexcel G4 = "z-statistic"

putexcel H4 = "p-value"

putexcel C5 = matrix(obl_mvprob), nformat(0.000)

putexcel close

putexcel set ${tables}plos_one_mvprob.xlsx, modify sheet(MVVoluntary)

putexcel B5 = "Female"

putexcel B6 = "Age"

putexcel B7 = "Tertiary education"

putexcel B8 = "Net monthly income per household, 1k EUR"

putexcel B9 = "Children younger than 17"

putexcel B10 = "Eastern federal states"

putexcel B11 = "Extraversion"

putexcel B12 = "Conscientiousness"

putexcel B13 = "Openness to experience"

putexcel B14 = "Neuroticism"

putexcel B15 = "Agreeableness"

putexcel B16 = "Willingness to take risks"

putexcel B17 = "Health: Self-assessment"

putexcel B18 = "Number of risk diseases"

putexcel B19 = "Test for COVID-19 in household"

putexcel B20 = "Prob. of life-threatening disease (in %)"

putexcel B21 = "Political preferences"

putexcel B22 = "Constant"

putexcel C4 = "Effect"

putexcel D4 = "S.E."

putexcel E4 = "LB 95% CI"

putexcel F4 = "UB 95% CI"

putexcel G4 = "z-statistic"

putexcel H4 = "p-value"

putexcel C5 = matrix(vol_mvprob), nformat(0.000)

putexcel close

putexcel set ${tables}plos_one_mvprob.xlsx, modify sheet(Obligatory)

putexcel B5 = "Female"

putexcel B6 = "Age"

putexcel B7 = "Tertiary education"

putexcel B8 = "Net monthly income per household, 1k EUR"

putexcel B9 = "Children younger than 17"

putexcel B10 = "Eastern federal states"

putexcel B11 = "Extraversion"

putexcel B12 = "Conscientiousness"

putexcel B13 = "Openness to experience"

putexcel B14 = "Neuroticism"

putexcel B15 = "Agreeableness"

putexcel B16 = "Willingness to take risks"

putexcel B17 = "Health: Self-assessment"

putexcel B18 = "Number of risk diseases"

putexcel B19 = "Test for COVID-19 in household"

putexcel B20 = "Prob. of life-threatening disease (in %)"

putexcel B21 = "Political preferences"

putexcel B22 = "Constant"

putexcel C4 = "Effect"

putexcel D4 = "S.E."

putexcel E4 = "LB 95% CI"

putexcel F4 = "UB 95% CI"

putexcel G4 = "z-statistic"

putexcel H4 = "p-value"

putexcel C5 = matrix(ob_prob), nformat(0.000)

putexcel close

putexcel set ${tables}plos_one_mvprob.xlsx, modify sheet(Voluntary)

putexcel B5 = "Female"

putexcel B6 = "Age"

putexcel B7 = "Tertiary education"

putexcel B8 = "Net monthly income per household, 1k EUR"

putexcel B9 = "Children younger than 17"

putexcel B10 = "Eastern federal states"

putexcel B11 = "Extraversion"

putexcel B12 = "Conscientiousness"

putexcel B13 = "Openness to experience"

putexcel B14 = "Neuroticism"

putexcel B15 = "Agreeableness"

putexcel B16 = "Willingness to take risks"

putexcel B17 = "Health: Self-assessment"

putexcel B18 = "Number of risk diseases"

putexcel B19 = "Test for COVID-19 in household"

putexcel B20 = "Prob. of life-threatening disease (in %)"

putexcel B21 = "Political preferences"

putexcel B22 = "Constant"

putexcel C4 = "Effect"

putexcel D4 = "S.E."

putexcel E4 = "LB 95% CI"

putexcel F4 = "UB 95% CI"

putexcel G4 = "z-statistic"

putexcel H4 = "p-value"

putexcel C5 = matrix(vol_prob), nformat(0.000)

putexcel close

putexcel set ${tables}plos_one_mvprob.xlsx, modify sheet(AvgVoluntary)

putexcel B5 = "Female"

putexcel B6 = "Age"

putexcel B7 = "Tertiary education"

putexcel B8 = "Net monthly income per household, 1k EUR"

putexcel B9 = "Children younger than 17"

putexcel B10 = "Eastern federal states"

putexcel B11 = "Extraversion"

putexcel B12 = "Conscientiousness"

putexcel B13 = "Openness to experience"

putexcel B14 = "Neuroticism"

putexcel B15 = "Agreeableness"

putexcel B16 = "Willingness to take risks"

putexcel B17 = "Health: Self-assessment"

putexcel B18 = "Number of risk diseases"

putexcel B19 = "Test for COVID-19 in household"

putexcel B20 = "Prob. of life-threatening disease (in %)"

putexcel B21 = "Political preferences"

putexcel C4 = "Effect"

putexcel D4 = "S.E."

putexcel E4 = "LB 95% CI"

putexcel F4 = "UB 95% CI"

putexcel G4 = "z-statistic"

putexcel H4 = "p-value"

putexcel C5 = matrix(vol_avgprob), nformat(0.000)

putexcel close

putexcel set ${tables}plos_one_mvprob.xlsx, modify sheet(AvgObligatory)

putexcel B5 = "Female"

putexcel B6 = "Age"

putexcel B7 = "Tertiary education"

putexcel B8 = "Net monthly income per household, 1k EUR"

putexcel B9 = "Children younger than 17"

putexcel B10 = "Eastern federal states"

putexcel B11 = "Extraversion"

putexcel B12 = "Conscientiousness"

putexcel B13 = "Openness to experience"

putexcel B14 = "Neuroticism"

putexcel B15 = "Agreeableness"

putexcel B16 = "Willingness to take risks"

putexcel B17 = "Health: Self-assessment"

putexcel B18 = "Number of risk diseases"

putexcel B19 = "Test for COVID-19 in household"

putexcel B20 = "Prob. of life-threatening disease (in %)"

putexcel B21 = "Political preferences"

putexcel B22 = "Constant"

putexcel C4 = "Effect"

putexcel D4 = "S.E."

putexcel E4 = "LB 95% CI"

putexcel F4 = "UB 95% CI"

putexcel G4 = "z-statistic"

putexcel H4 = "p-value"

putexcel C5 = matrix(ob_avgprob), nformat(0.000)

putexcel close

************************************************************

***** Comparing average marginal effects across models *****

************************************************************

use "$Data_prep\vaccination.dta", clear

rename Dum_voluntary out0

rename Dum_obligate out1

logit out1 ${righthand1}

margins, dydx(*) post

global righthand1 "D_female age education nettoy N_kids16 D_east extra consc open neuro agree risk health num_deseases_19 hh_test hh_positive p_severeill more_left"

global righthand2 "1.D_female age 1.education nettoy 1.N_kids16 1.D_east extra consc open neuro agree risk health num_deseases_19 1.hh_test p_severeill more_left"

# delimit ;

global righthand3 "i.obligate##i.D_female i.obligate##c.age i.obligate##i.education

i.obligate##c.nettoy i.obligate##i.N_kids16 i.obligate##i.D_east i.obligate##c.extra i.obligate##c.consc i.obligate##c.open i.obligate##c.neuro

i.obligate##c.agree i.obligate##c.risk i.obligate##c.health i.obligate##c.num_deseases_19 i.obligate##i.hh_test i.obligate##i.hh_positive i.obligate##c.p_severeill i.obligate##c.more_left";

# delimit cr

local num : list sizeof global(righthand2)

matrix mat_test = J(17,2,.)

keep pid out1 out0 ${righthand1}

reshape long out, i(pid) j(obligate)

local n = 0

logit out $righthand3, vce(robust)

margins, dydx(*) over(obligate) post

test [1.D_female]0.obligate = [1.D_female]1.obligate

mat mat_test[1,1] = r(chi2)

mat mat_test[1,2] = r(p)

test [age]0.obligate = [age]1.obligate

mat mat_test[2,1] = r(chi2)

mat mat_test[2,2] = r(p)

test [1.education]0.obligate = [1.education]1.obligate

mat mat_test[3,1] = r(chi2)

mat mat_test[3,2] = r(p)

test [nettoy]0.obligate = [nettoy]1.obligate

mat mat_test[4,1] = r(chi2)

mat mat_test[4,2] = r(p)

test [1.N_kids16]0.obligate = [1.N_kids16]1.obligate

mat mat_test[5,1] = r(chi2)

mat mat_test[5,2] = r(p)

test [1.D_east]0.obligate = [1.D_east]1.obligate

mat mat_test[6,1] = r(chi2)

mat mat_test[6,2] = r(p)

test [extra]0.obligate = [extra]1.obligate

mat mat_test[7,1] = r(chi2)

mat mat_test[7,2] = r(p)

test [consc]0.obligate = [consc]1.obligate

mat mat_test[8,1] = r(chi2)

mat mat_test[8,2] = r(p)

test [open]0.obligate = [open]1.obligate

mat mat_test[9,1] = r(chi2)

mat mat_test[9,2] = r(p)

test [neuro]0.obligate = [neuro]1.obligate

mat mat_test[10,1] = r(chi2)

mat mat_test[10,2] = r(p)

test [agree]0.obligate = [agree]1.obligate

mat mat_test[11,1] = r(chi2)

mat mat_test[11,2] = r(p)

test [risk]0.obligate = [risk]1.obligate

mat mat_test[12,1] = r(chi2)

mat mat_test[12,2] = r(p)

test [health]0.obligate = [health]1.obligate

mat mat_test[13,1] = r(chi2)

mat mat_test[13,2] = r(p)

test [num_deseases_19]0.obligate = [num_deseases_19]1.obligate

mat mat_test[14,1] = r(chi2)

mat mat_test[14,2] = r(p)

test [1.hh_test]0.obligate = [1.hh_test]1.obligate

mat mat_test[15,1] = r(chi2)

mat mat_test[15,2] = r(p)

test [p_severeill]0.obligate = [p_severeill]1.obligate

mat mat_test[16,1] = r(chi2)

mat mat_test[16,2] = r(p)

test [more_left]0.obligate = [more_left]1.obligate

mat mat_test[17,1] = r(chi2)

mat mat_test[17,2] = r(p)

regsave, ci pval tstat

drop if mi(tstat)

drop if var == "1.obligate:1.obligate" | var == "1.hh_positive:0bn.obligate"

preserve

* keep even observations

keep if mod(_n,2)

keep coef

mkmat _all, mat(comp_vol)

restore

preserve

* keep odd observations

keep if !mod(_n,2)

keep coef

mkmat _all, mat(comp_obl)

restore

putexcel set ${tables}plos_one_model_comparison.xlsx, modify sheet(comp)

putexcel B5 = "Female"

putexcel B6 = "Age"

putexcel B7 = "Tertiary education"

putexcel B8 = "Net monthly income per household, 1k EUR"

putexcel B9 = "Children younger than 17"

putexcel B10 = "Eastern federal states"

putexcel B11 = "Extraversion"

putexcel B12 = "Conscientiousness"

putexcel B13 = "Openness to experience"

putexcel B14 = "Neuroticism"

putexcel B15 = "Agreeableness"

putexcel B16 = "Willingness to take risks"

putexcel B17 = "Health: Self-assessment"

putexcel B18 = "Number of risk diseases"

putexcel B19 = "Test for COVID-19 in household"

putexcel B20 = "Prob. of life-threatening disease (in %)"

putexcel B21 = "Political preferences"

putexcel C4 = "Effect Vol."

putexcel D4 = "Effect Mand."

putexcel E4 = "Chi2(1)"

putexcel F4 = "p-value"

putexcel C5 = matrix(comp_vol), nformat(0.000)

putexcel D5 = matrix(comp_obl), nformat(0.000)

putexcel E5 = matrix(mat_test), nformat(0.000)

putexcel close

************************************************

********** Display of main results *************

************************************************

use "$Data_prep\vaccination.dta", clear

global righthand "i.D_female age i.education nettoy i.N_kids16 i.D_east extra consc open neuro agree risk health num_deseases_19 i.hh_test i.hh_positive p_severeill more_left"

logit Dum_voluntary $righthand, vce(robust)

margins, dydx(*) post

/*

Average marginal effects Number of obs = 678

Model VCE : Robust

Expression : Pr(Dum_voluntary), predict()

dy/dx w.r.t. : 1.D_female age 1.education nettoy 1.N_kids16 1.D_east extra consc open neuro agree risk health num_deseases_19 1.hh_test

1.hh_positive p_severeill more_left

---------------------------------------------------------------------------------

| Delta-method

| dy/dx Std. Err. z P>|z| [95% Conf. Interval]

----------------+----------------------------------------------------------------

1.D_female | -.1004701 .0352751 -2.85 0.004 -.169608 -.0313321

age | .0035352 .0012912 2.74 0.006 .0010046 .0060658

1.education | .1309117 .0358956 3.65 0.000 .0605576 .2012658

nettoy | .0251506 .0125314 2.01 0.045 .0005895 .0497117

1.N_kids16 | -.0040962 .0395224 -0.10 0.917 -.0815586 .0733662

1.D_east | .0016989 .0396695 0.04 0.966 -.0760519 .0794498

extra | -.0104519 .0172037 -0.61 0.543 -.0441705 .0232667

consc | -.0194879 .0180685 -1.08 0.281 -.0549016 .0159258

open | .0301721 .018319 1.65 0.100 -.0057324 .0660766

neuro | -.0243621 .0189544 -1.29 0.199 -.0615121 .0127878

agree | -.0209444 .0174234 -1.20 0.229 -.0550936 .0132048

risk | -.0250966 .0182811 -1.37 0.170 -.060927 .0107338

health | -.0057017 .0181041 -0.31 0.753 -.0411851 .0297817

num_deseases_19 | .0172114 .019058 0.90 0.366 -.0201415 .0545643

1.hh_test | -.0482994 .0509346 -0.95 0.343 -.1481294 .0515307

1.hh_positive | -.3189597 .3039058 -1.05 0.294 -.9146041 .2766848

p_severeill | .0026902 .0008271 3.25 0.001 .0010691 .0043113

more_left | .0120023 .0161017 0.75 0.456 -.0195564 .0435609

---------------------------------------------------------------------------------

Note: dy/dx for factor levels is the discrete change from the base level.

*/

logit Dum_obligate $righthand, vce(robust)

margins, dydx(*) post

/*

Average marginal effects Number of obs = 682

Model VCE : Robust

Expression : Pr(Dum_obligate), predict()

dy/dx w.r.t. : 1.D_female age 1.education nettoy 1.N_kids16 1.D_east extra consc open neuro agree risk health num_deseases_19 1.hh_test

1.hh_positive p_severeill more_left

---------------------------------------------------------------------------------

| Delta-method

| dy/dx Std. Err. z P>|z| [95% Conf. Interval]

----------------+----------------------------------------------------------------

1.D_female | -.0944152 .0394205 -2.40 0.017 -.171678 -.0171524

age | .0058068 .001476 3.93 0.000 .0029139 .0086997

1.education | -.0490708 .042111 -1.17 0.244 -.1316069 .0334653

nettoy | .0040743 .0107223 0.38 0.704 -.0169411 .0250897

1.N_kids16 | .0375643 .0463547 0.81 0.418 -.0532892 .1284179

1.D_east | .1444124 .0458967 3.15 0.002 .0544565 .2343683

extra | .0006698 .0189862 0.04 0.972 -.0365424 .037882

consc | .0139252 .0197537 0.70 0.481 -.0247913 .0526417

open | -.0014091 .0193168 -0.07 0.942 -.0392694 .0364512

neuro | -.0444538 .0201538 -2.21 0.027 -.0839545 -.004953

agree | -.002098 .0191342 -0.11 0.913 -.0396003 .0354042

risk | .009988 .0200726 0.50 0.619 -.0293535 .0493296

health | -.0023883 .0207525 -0.12 0.908 -.0430624 .0382858

num_deseases_19 | .0218824 .0204857 1.07 0.285 -.0182688 .0620335

1.hh_test | .0073016 .0579747 0.13 0.900 -.1063268 .12093

1.hh_positive | . (not estimable)

p_severeill | .0028127 .0008857 3.18 0.001 .0010768 .0045487

more_left | .0022004 .0183854 0.12 0.905 -.0338343 .0382352

---------------------------------------------------------------------------------

Note: dy/dx for factor levels is the discrete change from the base level.

*/
